# A comprehensive metabolomics investigation of hippocampus, serum, and feces affected by chronic fluoxetine treatment using the chronic unpredictable mild stress mouse model of depression

**DOI:** 10.1038/s41598-019-44052-2

**Published:** 2019-05-20

**Authors:** Jing Zhao, Yang-Hee Jung, Yan Jin, Seulgi Kang, Choon-Gon Jang, Jeongmi Lee

**Affiliations:** 1 School of Pharmacy, Sungkyunkwan University, Suwon, 16419 Republic of Korea; 2 Present Address: Shenyang Pharmaceutical University, Shenyang, 110016 P.R. China

**Keywords:** Predictive markers, Depression

## Abstract

A metabolomic investigation of depression and chronic fluoxetine treatment was conducted using a chronic unpredictable mild stress model with C57BL/6N mice. Establishment of the depressive model was confirmed by body weight measurement and behavior tests including the forced swim test and the tail suspension test. Behavioral despair by depression was reversed by four week-treatment with fluoxetine. Hippocampus, serum, and feces samples collected from four groups (control + saline, control + fluoxetine, model + saline, and model + fluoxetine) were subjected to metabolomic profiling based on ultra-high performance liquid chromatography-quadrupole-time-of-flight mass spectrometry. Alterations in the metabolic patterns were evident in all sample types. The antidepressant effects of fluoxetine appeared to involve various metabolic pathways including energy metabolism, neurotransmitter synthesis, tryptophan metabolism, fatty acid metabolism, lipid metabolism, and bile acid metabolism. Predictive marker candidates of depression were identified, including β-citryl-L-glutamic acid (BCG) and docosahexaenoic acid (DHA) in serum and chenodeoxycholic acid and oleamide in feces. This study suggests that treatment effects of fluoxetine might be differentiated by altered levels of tyramine and BCG in serum, and that DHA is a potential serum marker for depression with positive association with hippocampal DHA. Collectively, our comprehensive study provides insights into the biochemical perturbations involved in depression and the antidepressant effects of fluoxetine.

## Introduction

Depression is a debilitating condition that can have profound effects on both the mind and body of individuals who suffer from the disorder^1^. Globally, more than 300 million people of all ages suffer from depression. The World Health Organization has predicted that depression will be the second largest contributor to the global burden of disease by 2020^2^. Without treatment, depression can deteriorate significantly and even become life threatening. Antidepressant medications can relieve and resolve symptoms of depression. Among various drug classes currently available for treatment of depression, selective serotonin reuptake inhibitors (SSRIs) are generally prescribed for several forms of depression^3^, and fluoxetine is one of the most widely prescribed psychoactive SSRI pharmaceuticals^4^. Fluoxetine is absorbed well after oral administration, 6–8 h after which its plasma concentration reaches a peak. In general, SSRIs including fluoxetine can take several weeks to alleviate symptoms of depression in clinical patients^4^, and reasons for the delayed onset of therapeutic action remain unknown. There has been great demand for the development of novel antidepressants with rapid onset.

The chronic unpredictable mild stress (CUMS) model is a widely used rodent model of depression^5^. CUMS over a sustained period from 10 days to 8 weeks can establish a model that develops both behavioral and physiological abnormalities characteristic of human depression and is pharmacologically sensitive to a variety of antidepressant treatments^5^. Therefore, the CUMS model has been frequently employed for studying depression and diverse antidepressants including our previous metabolomics-based study^6–8^.

Metabolomics is the study of metabolism at the global level within cells or biological systems. Our previous metabolomic investigation of the hippocampus based on gas chromatography-mass spectrometry (GC-MS) provided insights into the molecular mechanisms of depression and revealed different biochemical changes induced by fluoxetine and imipramine under sub-chronic (two weeks) drug treatment in the CUMS mouse model^6^. Intriguingly, two-week treatment with fluoxetine failed to reverse the depression-like symptoms of the C57BL/6N strain in the forced swim test (FST), while it induced noticeable changes in the open field test (OFT) and body weight. The slow onset of action of fluoxetine in rodents and in the clinic^4^ led us to hypothesize that longer (chronic) treatment is needed to mitigate the symptoms of stress-related behaviors in the CUMS model with this mouse strain.

**Figure 1:**
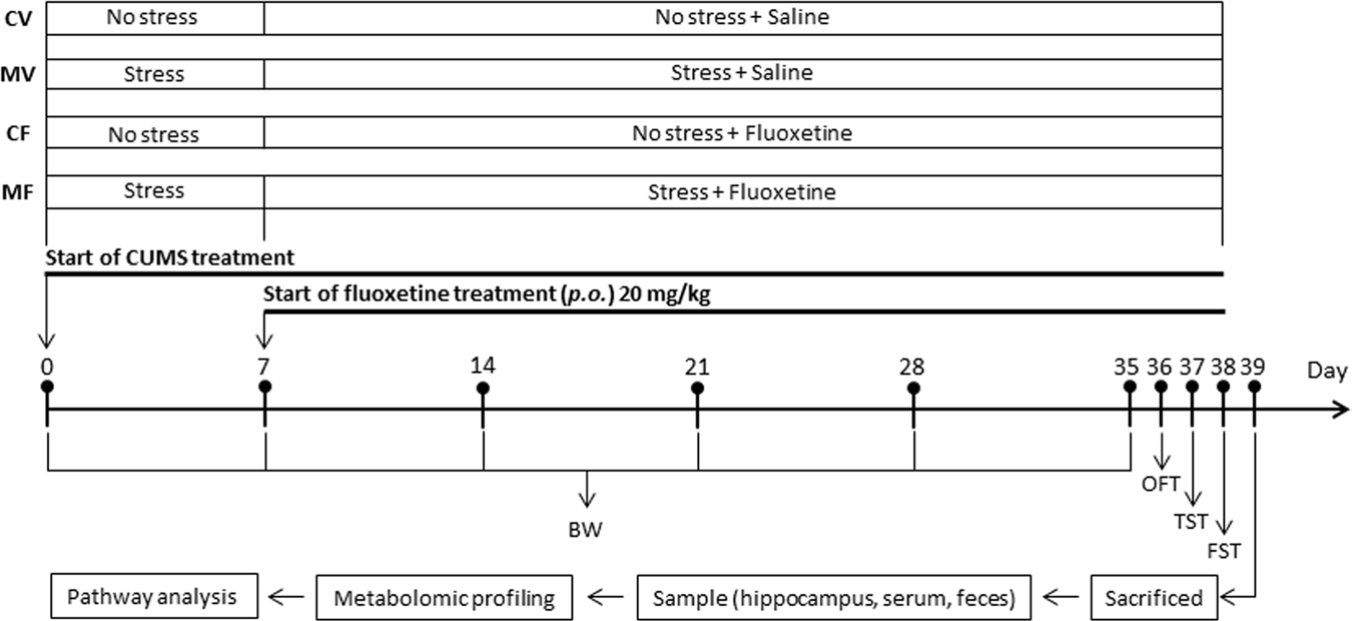
Schematic diagram of the experiment design.

In this study, we conducted a comprehensive metabolomics study to investigate the effects of chronic treatment of fluoxetine in the CUMS model with C57BL/6N mice. The experimental schematic is displayed in Fig. 1. The mice were classified into four groups depending on the stressors and fluoxetine treatment. The entire stress period was conducted for five weeks, with fluoxetine treatment administered for the last four weeks. After body weight measurements and behavior tests including OFT, FST, and tail suspension test (TST) were conducted, various sample types were subjected to metabolomics analysis. Hippocampus, a relevant brain region for studying depression and antidepressant effects^2^, and minimally or non-invasive samples including serum and feces were included because they could be informative and useful for the discovery of biomarkers indicating the development of depression or treatment efficacy. Metabolic profiling was performed using ultra-high performance liquid chromatography coupled to quadrupole-time-of-flight-mass spectrometry (UHPLC-Q-TOF-MS), one of the premier analytical platforms for metabolomics studies, and the intervention mechanism of fluoxetine for depression was identified by analyzing metabolic pathways and networks. To the best of our knowledge, this is the first comprehensive metabolomics study of biochemical changes in hippocampus, serum, and feces by chronic treatment with fluoxetine using the CUMS mouse model.

## Results and Discussion

### Alterations in behavior test results and body weight by stressors and fluoxetine

**Figure 2:**
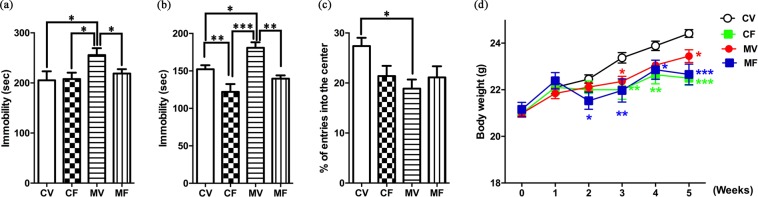
Behavior tests and body weight measurement. (**a**) FST, (**b**) TST, (**c**) OFT, (**d**) body weight. CV, control treated with saline; CF, control treated with fluoxetine; MV, CUMS model treated with saline; MF, CUMS model treated with fluoxetine. Error bars indicate the SEM (n = 7). Statistical analysis was performed using one-way ANOVA (FST, TST, and OFT) and two-way ANOVA (body weight), which were followed by Tukey’s multiple comparison test and Fisher’s LSD test, respectively. (**a**–**c**) *, **, and *** indicate *p* < 0.05, *p* < 0.01, and *p* < 0.001, respectively, for a given pair. (**d**) *, **, and *** indicate *p* < 0.05, *p* < 0.01, and *p* < 0.001, respectively, in comparison with CV.

FST is one of the most commonly used tools to screen antidepressants, and TST is another well-established screening paradigm, frequently used to determine depression- and antidepressant-like behaviors in rodents after exposure to various stressors^8^. Immobility time is a vital endpoint of the CUMS model as an indication of helpless behavior in both FST and TST^9^. Due to tremendous amount of stress from the tests, FST and TST were measured only once at the end of the CUMS period. Specifically, after OFT on day 36, TST was conducted on day 37, followed by FST on day 38 (Fig. 1). Because immobility time can be measured under different time periods and settings of video tracking system, its values of control groups have been reported in a wide range even within the same mouse strain. For example, they were as low as ~90 s^10^ and ~75 s^11^ and as high as ~180 s^11^ and ~220 s^12^ for TST and FST, respectively. The measured values of the control mice (CV) in Fig. 2 were relatively large (151 s in TST and 208 s in FST); however, the immobility time was significantly increased in the MV group in both tests (F_1,12_ = 7.883, 0.019 for TST; F_1,12_ = 7.832, 0.016 for FST), indicating that the CUMS model of depression had been successfully established^13^.

In FST, fluoxetine treatment resulted in a significantly reduced immobility time in the stressed mice (MF *vs*. MV; F_1,12_ = 18.740, <0.001), while it exerted no discernible effects in the unstressed groups (CF *vs*. CV). In TST (Fig. 2b), fluoxetine significantly lowered the immobility time in both stressed (MF *vs*. MV; F_1,12_ = 12.628, 0.005) and unstressed (CF *vs*. CV; F_1,12_ = 6.405, 0.029) mice. The reduced immobility times by chronic fluoxetine treatment (4 weeks) in both tests indicate that fluoxetine successfully reversed the depression-like symptoms. This result is different from our previous study showing that subchronic treatment of fluoxetine (2 weeks) failed to alter the immobility time of stressed mice in FST^6^. These findings suggest that the CUMS model of C57BL/6N mice requires a long duration of fluoxetine treatment to exhibit an antidepressant effect, which is similar to clinical findings^4^. However, the results of the two behavioral despair tests were not in complete agreement, as the TST results showing a significantly decreased immobility time by fluoxetine in the unstressed mice are consistent with the literature^13–15^. Although the underlying mechanisms for different responses in the two behavior tests remain to be clarified, it is likely that they are mediated by different neurobiological pathways^16^. Our results suggest that TST might be more useful than FST for evaluating antidepressant effects using C57BL/6N mice without application of stressors.

OFT has been used to measure locomotor activity and emotionality from exploration and anxiety in rodents. OFT was conducted at the end of the stress period. No significant differences were observed in the total distance travelled (data not shown) among the four groups; however, the percent of entries into the center (% entries) of the CV group was significantly higher than that of the MV group (Fig. 2c; F_1,12_ = 11.984, 0.005). Stressors significantly lowered the center entries, and fluoxetine did not reverse the number of center entries in the stressed groups (MF *vs*. MV; F_1,12_ = 0.606, 0.452). The results are partially inconsistent with chronic fluoxetine treatment having a sedative effect (diminution in both center entries and total locomotor activity) in C57BL/6 mice^17^. In fact, fluoxetine effects on OFT results have been controversial. These inconsistencies are possibly due to differences in experimental settings including strain, dosage, administration method, treatment period, and OF environment in addition to those in measured parameters^17^.

Body weight changes were monitored weekly during the CUMS procedure (Fig. 2d). The MV group showed significantly lower body weights than the CV group between the third and fifth weeks (F_1,12_ = 9.205, 0.010), which implies possible food intake reduction caused by stressors, as is found in human patients with depression^18^. While CV mice showed continuous body weight gain for five weeks, the fluoxetine-treated mice (both CF and MF) underwent significant weight loss from the third week. At the end of the stress period, the body weights of the fluoxetine-treated control mice were lower than those of MV mice (CF *vs*.MV, F_1,12_ = 5.202, 0.042), but there was no significant difference between MF and MV mice (F_1,12_ = 2.268, 0.158). These results, which imply food intake suppression by fluoxetine, are consistent with studies on rodents^19,20^ and humans^18^, but not with our previous observations^6^. This discrepancy appears to be associated with the different administration routes; *i.p*. injection might have mitigated the effects of fluoxetine on appetite, because weight loss by SSRIs is associated with a decrease in appetite^21^.

### Metabolomic profiling of three different types of samples

According to the literature^22,23^ and our own experiments that acetonitrile, methanol, 70% acetonitrile, and 70% methanol were compared, methanol was selected as the extraction solvent because it allowed for simple, rapid, and consistent extraction of metabolites with various polarities regardless of sample type and was easy to remove after extraction. Extraction was facilitated by ultrasonic irradiation in ice bath for handling numerous samples simultaneously with ease. Depending on the sample type, different chromatographic conditions were established for enhanced peak resolution and ionization of metabolites from a wide variety of polarities within a short time. The experimental conditions are described in the Materials and methods section.

Principal component analysis (PCA) was used to visualize general clustering, trends, or outliers among the observations acquired in positive (POS) and negative (NEG) modes (Fig. S1). Quality control (QC) samples were analyzed to monitor the stability of the analytical system and were found to cluster closely in the score plots of all three sample types (Fig. S2). The key parameters, R^2^ and Q^2^, were used for the evaluation of discrimination and predictive abilities of the models, respectively. They are shown in Table S1, suggesting that all models were robust and had good fitness and prediction. One sample (CF 6) in the CF group was detected as a potential outlier in the plot of hippocampus in POS mode (Fig. S1a). However, it was located inside the boundary in the plot of NEG mode (Fig. S1b), and Hotelling’s T2 plots exhibited no outliers (data not shown). Accordingly, all of the data were included for further multivariate and univariate analyses.

It was notable that the CV group was distinctively differentiated from the other three groups in both POS and NEG mode analyses of hippocampus samples (Figs S1a,b). In addition, the serum samples analyzed in NEG mode showed a clear distinction between the vehicle-treated (CV and MV) groups and the fluoxetine-treated groups (CF and MF) (Fig. S1d).

### Identification of differential metabolites through multivariate statistical analysis

**Figure 3:**
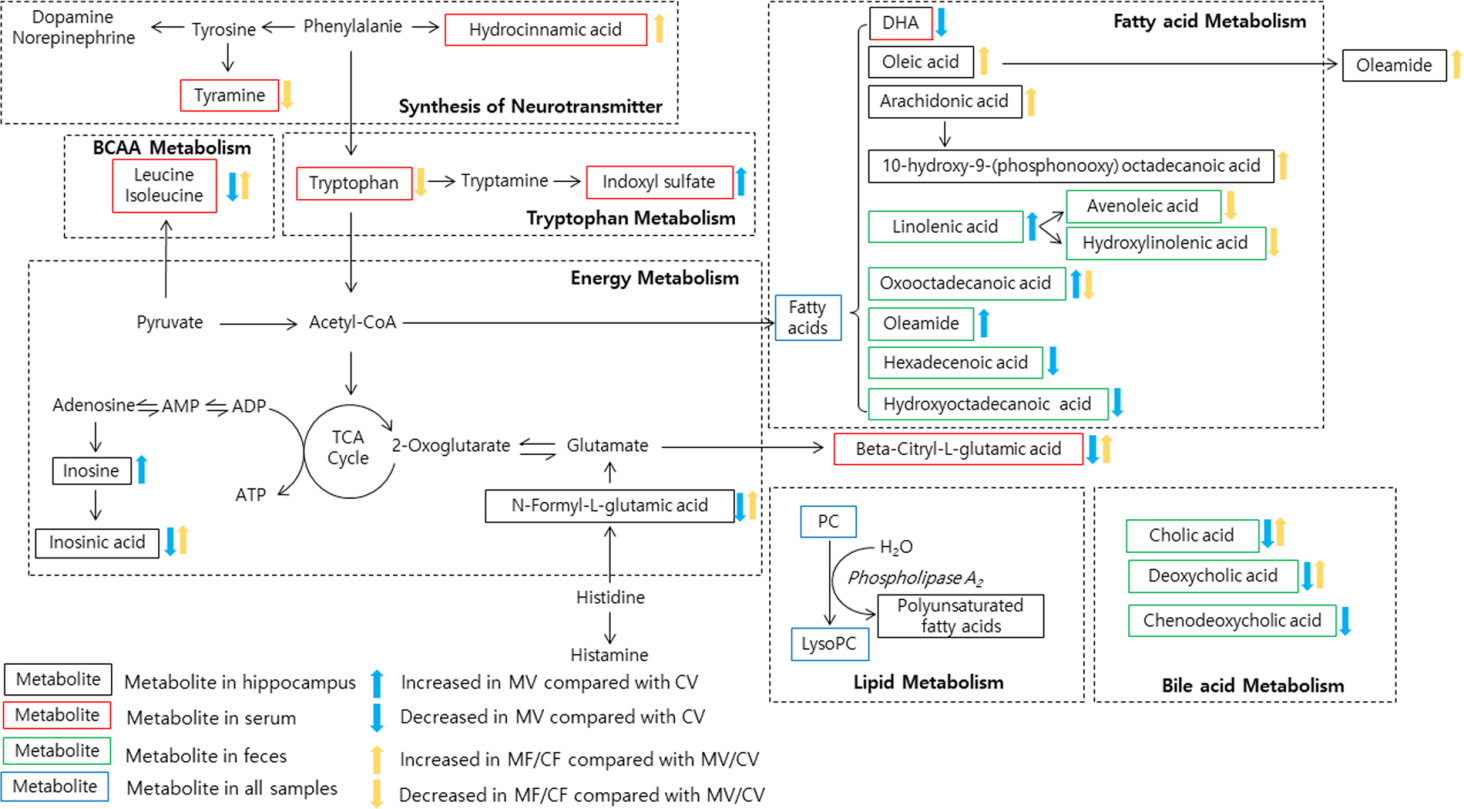
Metabolic pathways affected by CUMS and chronic treatment with fluoxetine.

To better understand the metabolic perturbations induced by stressors and/or fluoxetine, a supervised multiple regression analysis, pair-wise orthogonal projections to latent structures discriminant analysis (OPLS-DA), was applied to each kind of sample. The parameter R^2^Y indicates the total explained variation for the X matrix, and Q^2^ represents the predictability of the model. Eighteen OPLS-DA models were built, and the related parameters of R^2^Y and Q^2^ were greater than 0.5 (Table S2), which indicated the models were stable with a reliable predictive ability. The OPLS-DA score plots of samples analyzed in POS and NEG modes are displayed in Figs S3–S5. Clear separation was observed for all of the pair-wise comparisons (CV *vs*. MV, MV *vs*. MF, and CV *vs*. CF) with all three sample types. Differential metabolites were selected from the V-plot that was constructed using the variable importance in projection (VIP) value *vs*. coefficient of each variable. Metabolites for which the absolute values of VIP score were larger than 1 and coefficients of metabolites far from the center were selected (Figs S6–S8) and further confirmed by S-plot and Student’s *t*-test to decrease the risk of false positives in the marker selection (data not shown). The identified differential markers from the three sample types are listed in Tables 1, 2 and 3. Based on the extracted differential metabolites, pathway analysis was conducted, and the results are summarized in Fig. 3.

**Table 1:** List of differential metabolites for discrimination among the CV, MV, CF, and MF groups from hippocampal analysis.

t_R_ (min)	Metabolite	Formula	Ionization mode	Measured *m/z*	Mass error (mDa)	MV *vs*. CV		MF *vs*. MV		CF *vs*. CV	
						Fold change	VIP score	Fold change	VIP score	Fold change	VIP score
0.73	N-Formyl-L-glutamic acid	C_6_H_9_NO_5_	NEG	174.0396	−0.6	0.90	4.2	2.91	2.6	1.13	4.4
0.82	Inosinic acid	C_10_H_13_N_4_O_8_P	NEG	347.0400	0.7	0.26	2.6	2.57	3.3	1.64	2.8
0.91	Glutathione	C_10_H_17_N_3_O_6_S	NEG	306.0763	0.3	0.55	2.1	2.53	3.9		
1.10	Inosine	C_10_H_12_N_4_O_5_	NEG	267.0728	−0.1	1.33	4.4	1.15	2.8		
4.23	Docosahexaenoic acid	C_22_H_32_O_2_	NEG	327.2321	−0.3	0.87	3.1				
4.80	LysoPC(16:0)	C_24_H_50_NO_7_P	POS	496.3404	0.1					1.23	11.3
4.87	LysoPE(18:1/0:0)	C_23_H_46_NO_7_P	NEG	478.2934	0.0	0.73	7.3	3.93	3.9	4.89	3.9
5.81	Oleamide	C_18_H_35_NO	POS	282.2796	−0.1			2.50	7.5		
6.10	(9S, 10S)-10-Hydroxy-9-(phosphonooxy)octadecanoic acid	C_18_H_37_O_7_P	NEG	395.2204	0.5			1.40	6.1		
6.19	Arachidonic acid	C_20_H_32_O_2_	NEG	303.2322	−0.2					1.06	3.4
6.20	LysoPC(18:0)	C_26_H_54_NO_7_P	POS	524.3717	0.1	0.85	6.8	1.17	5.2	1.18	6.9
7.49	Oleic acid	C_18_H_34_O_2_	NEG	281.2472	−0.9			1.50	6.2		
7.81	MG(18:0/0:0/0:0)	C_21_H_42_O_4_	POS	359.3157	−0.4	0.07	18.7	17.65	19.4	1.51	12.5
14.92	PC(14:0/18:1)	C_40_H_78_NO_8_P	POS	732.5549	0.6					1.59	10.3
16.16	3-O-Sulfogalactosylceramide (d18:1/24:1)	C_48_H_91_NO_11_S	NEG	888.6232	−0.3	0.82	3.5				
16.69	PE(20:3/P-18:1)	C_43_H_78_NO_7_P	NEG	750.5427	−1.1			0.31	3.0		
18.36	PC(18:0/22:1)	C_48_H_94_NO_8_P	NEG	842.6720	8.1			0.53	2.9		
18.60	PC(o-22:1/20:4)	C_50_H_92_NO_7_P	POS	850.6753	−1.1	1.18	7.7

**Table 2:** List of differential metabolites for discrimination among the CV, MV, CF, and MF groups from serum analysis.

t_R_ (min)	Metabolite	Formula	Ionization mode	Measured *m/z*	Mass error (mDa)	MV *vs*. CV		MF *vs*. MV		CF *vs*. CV	
						Fold change	VIP score	Fold change	VIP score	Fold change	VIP score
1.22	L-Leucine/L-Isoleucine	C_6_H_13_NO_2_	POS	132.1008	−1.7	0.79	10.0			1.72	7.4
1.41	Tyramine^a^	C_8_H_11_NO	POS	160.0747	0.9			0.04	11.9	0.04	14.8
2.00	L-Tryptophan	C_11_H_12_N_2_O_2_	POS	205.0965	−1.2					0.66	9.7
			NEG	203.0822	0.1					0.42	4.5
2.48	Indoxyl sulfate	C_4_H_8_NO_7_P	NEG	212.0016	5.6	2.61	7.17				
3.90	5-Thymidylic acid	C_10_H_15_N_2_O_8_P	NEG	321.0432	−5.6	2.07	6.03				
11.73	β-Citryl-L-glutamic acid^b^	C_11_H_15_NO_10_	NEG	641.1373	5.9	0.57	5.41	237.34	9.0	103.00	9.9
11.74	LysoPC(16:1)	C_24_H_48_NO_7_P	POS	494.3240	−0.7	0.59	13.0			2.74	6.2
12.48	LysoPE(0:0/18:2)	C_23_H_44_NO_7_P	POS	478.2922	−1.2	1.69	12.4	1.28	6.7		
			NEG	476.2781	0.4	1.52	6.12			0.56	3.8
12.59	LysoPE(0:0/20:2)	C_25_H_48_NO_7_P	NEG	504.3099	0.9	1.41	11.77	0.78	7.7	0.80	3.5
12.59	LysoPC(18:2)	C_26_H_50_NO_7_P	POS	520.3408	0.5	1.18	29.7			0.93	13.3
12.68	LysoPC(20:4)	C_28_H_50_NO_7_P	POS	544.3404	0.1	0.63	23.3	0.58	14.5	0.81	12.8
12.68	LysoPE(0:0/22:4)	C_27_H_48_NO_7_P	NEG	528.3098	0.8			0.54	5.0		
13.40	LysoPC(15:0)	C_23_H_48_NO_7_P	NEG	480.3085	−0.5			0.66	10.0	0.70	9.6
13.40	LysoPC(16:0)	C_24_H_50_NO_7_P	POS	496.3403	0.0			0.34	23.9		
13.51	LysoPC(20:3)	C_28_H_52_NO_7_P	POS	546.3554	−0.6	0.77	8.4				
14.10	LysoPE(0:0/20:1)	C_25_H_50_NO_7_P	NEG	506.3244	−0.3	0.71	10.52				
14.10	LysoPC(18:1)	C_26_H_52_NO_7_P	POS	522.3551	−0.9			0.60	20.6	0.76	17.7
15.89	Hydrocinnamic acid^c^	C_9_H_10_O_2_	POS	301.1401	−3.9			1.53	6.4	1.48	11.4
19.24	Docosahexaenoic acid	C_22_H_32_O_2_	NEG	327.2317	−1.6	0.37	4.78				

^a^[M+Na]^+^.

^b^[2M-H]^−^.

^c^[2M+H]^+^.

**Table 3:** List of differential metabolites for discrimination among the CV, MV, CF, and MF groups from feces analysis.

t_R_ (min)	Metabolite	Formula	Ionization mode	Measured *m/z*	Mass error (mDa)	MV *vs*. CV		MF *vs*. MV		CF *vs*. CV	
						Fold change	VIP score	Fold change	VIP score	Fold change	VIP score
6.27	Adrenic acid^a^	C_22_H_36_O_2_	POS	355.2612	−0.1					1.50	3.2
6.27	Cholic acid	C_24_H_40_O_5_	NEG	407.2795	−0.2	0.46	3.0	1.96	4.2	1.80	6.9
6.28	Cervonoyl ethanolamide	C_24_H_36_O_3_	POS	373.2711	−3.2			2.95	2.4	1.58	3.4
8.45	Chenodeoxycholic acid	C_24_H_40_O_4_	NEG	391.2851	0.3	0.10	2.0				
9.81	3-Oxo-4,6-choladienoic acid	C_24_H_34_O_3_	POS	371.2554	−3.2	1.76	3.0				
11.13	Deoxycholic acid	C_24_H_40_O_4_	NEG	391.2851	0.3	0.41	7.8	2.88	8.9		
	Deoxycholic acid^b^	C_24_H_40_O_4_	POS	785.5880	−5.1	0.15	4.6	5.49	3.2		
11.47	Ceanothenic acid	C_29_H_42_O_4_	POS	455.3160	−0.1	0.33	3.0	2.45	2.5		
11.62	LysoPE(0:0/18:2)	C_23_H_44_NO_7_P	NEG	476.2789	1.2			5.72	2.0		
12.98	1-Palmitoylglycerophosphoinositol	C_25_H_49_O_12_P	NEG	571.2891	0.8			0.70	2.5		
13.17	LysoPE(0:0/18:1)	C_23_H_46_NO_7_P	NEG	478.2919	−1.5					3.06	2.7
13.41	Avenoleic acid	C_18_H_32_O_3_	NEG	295.2253	−2.0			0.49	1.5		
13.60	N-Decanoylglycine^c^	C_12_H_23_NO_3_	NEG	457.3309	3.1	0.35	2.5			0.42	2.9
13.72	Oxooctadecanoic acid	C_18_H_34_O_3_	NEG	297.2419	−1.1	1.29	2.7	0.84	1.9		
13.84	Hydroxylinolenic acid	C_18_H_30_O_3_	NEG	293.2103	−1.4					0.50	5.3
13.85	Hexadecenoic acid^a^	C_16_H_30_O_2_	POS	277.2149	0.6	0.62	3.1				
14.08	LysoPE(0:0/16:0)	C_21_H_44_NO_7_P	POS	454.2897	−3.7					0.76	3.4
14.17	LysoPC(16:0)	C_24_H_50_NO_7_P	POS	496.3380	−2.3			0.71	5.0	0.67	6.4
14.80	LysoPC(18:1)	C_26_H_52_NO_7_P	POS	522.3534	−2.6	1.27	7.6	0.64	10.7	0.73	4.6
14.81	LysoPE(0:0/20:1)	C_25_H_50_NO_7_P	NEG	506.3223	−2.4			0.67	1.7	0.44	2.8
15.92	Hydroxyoctadecanoic acid	C_18_H_36_O_3_	NEG	299.2570	−1.6	0.52	3.1				
17.35	Linolenic acid	C_18_H_30_O_2_	NEG	277.2141	−2.7	1.31	2.7				
17.36	MG(0:0/18:2/0:0)	C_21_H_38_O_4_	POS	377.2628	−4.0	1.53	2.1				
17.65	Palmitic amide	C_16_H_33_NO	POS	256.2602	−3.8	2.59	3.0				
17.85	3a,7a-Dihydroxy-5b-cholestane^a^	C_27_H_48_O_2_	POS	427.3551	−0.1					0.78	3.5
18.00	Oleamide	C_18_H_35_NO	POS	282.2767	−3.0	4.44	4.6				

^a^[M+Na]^+^.

^b^[2M+H]^−^.

^c^[2M-H]^+^.

### Interpretation of potential marker metabolites

#### Fatty acid metabolism

Numerous fatty acids and related metabolites were differentially regulated by depression and fluoxetine. In hippocampus, oleic acid and oleamide were up-regulated by fluoxetine treatment (Table 1). Oleic acid, a monounsaturated omega-9 fatty acid, was previously shown to be up-regulated in the hippocampus with imipramine treatment^6^ and was associated with a reduced risk of severe depression in humans in an earlier 10-year follow-up study^24^. Treatment with oleamide, an amide of oleic acid, reversed the CUMS-induced depressive-like symptoms with differential expression of several key hippocampal proteins in a CUMS rat model^25^. Significantly elevated levels of oleic acid and oleamide in the hippocampus by fluoxetine treatment in our CUMS model support their antidepressant-like properties^26^.

Interestingly, docosahexaenoic acid (DHA) level was lowered in response to stressors in both hippocampus and serum (Tables 1 and 2), while it was not altered by fluoxetine treatment. DHA, an omega-3 fatty acid, is a primary structural component of brain, and preclinical studies indicated that DHA improves memory^27,28^. The decreased DHA level in stressed hippocampus might be associated with memory loss, which is one of the most characteristic symptoms of depression. Our results imply that decreased DHA level in the hippocampus might have an association with lowered DHA level in serum; a possible correlation was observed in the DHA levels between the plasma total lipids and brain phosphatidylethanolamine^29^. DHA might not be directly associated with the treatment effect of fluoxetine, but might be a predictive marker for depression in serum.

The amount and composition of fecal fatty acids can reflect fat ingestion, intestinal fatty acid absorption, and activity of colonic bacteria^30^. Similar to Yu *et al*.’s study using a rat model of depression^31^, fatty acid metabolism was markedly disturbed by the CUMS and fluoxetine treatment in our mouse model. It is likely that depression affected the digestion system^32^ as well as gut microbiota and fecal metabolome. A comprehensive study of gut microbiome will be needed to provide more insight into association of digestion system with depression and antidepressants.

#### Lipid metabolism

Disturbance of lipid metabolism by stressors and fluoxetine was observed in hippocampus, serum, and feces. Numerous publications have reported alterations in lipid profiles in association with depression; however, there are extensive variations and discrepancies^33–35^. In the present study, changes in lipid patterns by depression or fluoxetine differed depending on the sample type in general.

In the hippocampus (Table 1), the MG(18:0/0:0/0:0) level was severely affected by depression (~14-fold decrease) and fluoxetine treatment (~18-fold increase). MG(18:0/0:0/0:0) is a monoacylglycerol (MAG) that is broken down by MAG lipase (MAGL). 2-Arachionoylglycerol (2-AG), a unique MAG functioning as an endocannabinoid, was dysregulated in human and animal models of depression, and a selective MAGL inhibitor, JZL184, which inhibits degradation of 2-AG, exhibited antidepressant-like effects^36^. Thus, the antidepressant effects of fluoxetine might be associated with pathways involving MAGL.

Eleven lipid metabolites, seven lysophosphatidylcholines (lysoPCs), and four lysophosphatidylethanolamines (lysoPEs) were identified as differential metabolites in serum (Table 2). LysoPCs are not only the product of PCs that maintain the normal integrity of cell membranes^37^, but are also vital cell-signaling molecules^38^. Polyunsaturated lysoPCs are predominantly produced by phospholipase A_2_ (PLA_2_), while saturated lysoPCs are mostly produced by lecithin:cholesterol acyltransferase (LCAT)^39^. Depression has been characterized by oxidative stress, which causes an increase in the hepatic activity of PLA_2_ and hepatic hydrolysis of PC to elevate plasma concentrations of polyunsaturated lysoPCs^40^. Significantly decreased serum levels of lysoPCs such as lysoPC(16:1) and lysoPC(20:3) might be attributed to low serum LCAT activity, which was reported in depressive patients^41^. Levels of lysoPEs were generally lowered in response to fluoxetine treatment.

Levels of several fecal lipids including lysoPC(18:1) and MG(0:0/18:2/0:0) were elevated by stressors (Table 3). Fecal lysoPCs originate mainly from hydrolysis of biliary and dietary phosphatidylcholines, which are major components of biliary and dietary phospholipids, possibly due to gut microflora or PLA_2_^42^.

These results suggest that each type of sample has its own regulation of lipid metabolism. A detailed and comprehensive lipidomics study will be needed to provide deeper insight into lipid metabolism related to depression and antidepressant effects.

#### Amino acid metabolism and related pathways

N-formyl-L-glutamic acid is an intermediate in the metabolism of histidine and a precursor to histamine. It is also a precursor to L-glutamic acid, which was down-regulated by fluoxetine in the hippocampus in our previous study^6^. Its increase in the hippocampus by fluoxetine treatment, shown in Table 1, implies that fluoxetine treatment effects might be associated with the histaminergic neuron^43^ and glutamatergic systems^44^ or with the stimulated energy metabolism in hippocampus, of which non-synaptic mitochondria exhibited enhanced enzymatic activities including glutamate dehydrogenase^45,46^.Inosinic acid or ionosine monophosphate is formed by deamination of adenosine monophosphate and can be hydrolyzed to produce inosine. Inosine had an antidepressant-like effect in mice, as observed by FST^47^ and TST^48^, which is related to our results that inosinic acid in the hippocampus was significantly decreased by the CUMS treatment, and that fluoxetine treatment induced its up-regulation in both the model and control mice.

Glutathione (GSH) or γ-L-glutamyl-L-cysteinylglycine is considered the brain’s primary antioxidant and a measure of oxidative stress status in tissues^49^. The hippocampal GSH level was remarkably decreased in the MV mice compared with the CV mice, while it was up-regulated by fluoxetine in the CUMS mice (Table 1). This result is analogous to Jeremy *et al*.’s study showing decreased GSH in the post-mortem prefrontal cortex of patients with psychiatric disorders. Similarly, the GSH level in the brain was significantly elevated after treatment with escitalopram (SSRI class) in the CUMS model^50^. The decreased GSH level might have caused GSH deficiency in the MV mice that could lead to a variety of influences including increased oxidative stress and reduced detoxification ability.

Indoxyl sulfate, a metabolite of tryptophan, was up-regulated in the serum of depressed mice, while remaining unaffected by fluoxetine in the control and model mice. Together with DHA, it might serve as a predictive serum marker for depression. Tryptophan is a biochemical precursor of serotonin that plays a vital role in depression^51^. A significant decrease in tryptophan by fluoxetine treatment in the unstressed mice was shared in the serum analyses performed in both positive and negative modes (Table 2). These results imply that depression might be associated with abnormality in tryptophan metabolism, consistent with recent metabolomics studies in rats and humans^52,53^, and suggest that tryptophan metabolism is affected by fluoxetine intervention.

Leucine or isoleucine was differentially expressed in serum, with decreases observed in stressed mice and increases observed in fluoxetine-treated mice. Previously, the hippocampal leucine level was shown to be elevated by fluoxetine treatment^6^, and the concentrations of branched chain amino acids (BCAAs) increased upon paroxetine treatment, with leucine suggested as a marker candidate for antidepressant effects^54^. BCAAs can reduce central fatigue and also are directly related to energy metabolism. They also can compete with tryptophan, the level of which was down-regulated by fluoxetine treatment in this study.

A derivative of glutamic acid, β-citryl-L-glutamic acid (BCG), was down-regulated in the serum of CUMS model mice, and its level soared upon fluoxetine treatment in both the stressed and unstressed groups (Table 2). Since its first detection in the brain of new-born rats, physiological roles of BCG have remained largely unknown for decades. BCG was suggested as a substrate for glutamate carboxypeptidase III (GCPIII), a homologue of GCPII that is a protease involved in neurological disorders^55^. The current study suggests BCG as a potential predictive marker for depression and fluoxetine treatment effects.

#### Bile acid metabolism

Bile acids are not only important for lipid absorption and cholesterol homeostasis, but also play an important role in energy and glucose homeostasis^56^. The fecal metabolomic investigation revealed alterations in bile acid metabolism in the depressed mice (Table 3 and Fig. 3). Cholic acid, deoxycholic acid, and chenodeoxycholic acid levels were significantly decreased by stressors, and levels of the first two were elevated by fluoxetine treatment. In particular, deoxycholic acid was suggested as a potential marker for depression and treatment effect, given that it consistently showed pronounced reduction (2.4–6.7-fold) and elevation (2.9–5.5-fold) by stressors and fluoxetine treatment, respectively in both NEG and POS modes of detection. Cholic acid and chenodeoxycholic acid are major primary bile acids, while deoxycholic acid is a secondary bile acid that is a metabolic byproduct of intestinal bacteria^57^. The microbiome is involved in neurological functions and can affect mood and behavior through different pathways^58,59^.

## Conclusions

A UHPLC-Q-TOF-MS-based metabolomics study was conducted using the CUMS model of depression. Different behaviors and metabolic patterns in the hippocampus, serum, and feces were induced by depression and fluoxetine treatment. The behavioral despair test results suggest that the CUMS model of C57BL/6N mice requires chronic treatment of fluoxetine to exhibit antidepressant effects, and that TST could be desirable to evaluate antidepressant effects in mice without tedious application of stressors. The antidepressant effects of fluoxetine appear to involve various metabolic pathways including energy metabolism, synthesis of neurotransmitters, tryptophan metabolism, fatty acid metabolism, lipid metabolism, and bile acid metabolism. Numerous predictive marker candidates of depression were identified including indoxyl sulfate, BCG, and DHA in serum and deoxycholic acid, chenodeoxycholic acid, and oleamide in feces. Treatment effects of fluoxetine might be differentiated by altered levels of tyramine and BCG in serum or deoxycholic acid in feces. DHA might be a potential serum marker for depression that is positively associated with hippocampal DHA.

Collectively, our comprehensive study on hippocampus, serum, and feces using the CUMS model of depression suggests that differential markers provide insights into the metabolic pathways involved in depression and antidepressant effects of fluoxetine.

## Materials and Methods

### Chemicals and instruments

Ammonium formate and chlorpropamide of analytical grade were obtained from Sigma-Aldrich (St. Louis, MO, USA). Fluoxetine of analytical grade was purchased from TCI (Tokyo, Japan). HPLC-grade formic acid was obtained from Sigma-Aldrich, while HPLC-grade acetonitrile, methanol, and water were from J.T. Baker (Center Valley, PA, USA). Double-distilled water was prepared using a Milli-Q water purification system from Millipore (Bedford, MA, USA). Gyrozen centrifuge (Incheon, Korea) and ultrasonic bath (Ilshin, Korea) were used for centrifugation and ultrasonic extraction, respectively.

### Animals

Eight-week-old male C57BL/6N mice were purchased from Daehan Biolink Co., Ltd (Eumseong, Korea). After arrival, mice were acclimatized for one week prior to use in experimental procedures. Mice were housed four per cage and maintained in a temperature- and humidity-controlled room (23 ± 1 °C, 55 ± 5%) under a 12 h light/dark cycle (lights on at 07:00–19:00) with access to food and water *ad libitum* before applying the CUMS procedure. All animal care procedures were conducted in accordance with the US National Institutes of Health Guide for the Care and Use of Laboratory Animals and were approved by the Institutional Animal Care and Use Committee of Sungkyunkwan University.

### Procedures for chronic unpredicted mild stress (CUMS) and drug administration

Mice were randomly divided into four groups (n = 7 per group): control group treated with saline (CV), control group treated with fluoxetine (CF), CUMS model group treated with saline (MV), and CUMS model group treated with fluoxetine (MF). Mice in the CV and CF groups were housed in groups (three or four per cage), and mice in the MV and MF groups were singly housed. The average body weight of the mice was 20.2 g, showing no significant differences among the groups (*p* > 0.05) immediately after grouping.

The CUMS procedure consisted of a variety of unpredictable mild stressors including tilt cage, confinement, soiled bedding, white noise, removal of nesting materials, paired housing, reversed light dark cycle, and overnight illumination^6^. The stressors were presented to mice in a random order twice per day, in the morning (9:00) and in the evening (19:00), from day 1 to day 35. From day 8 to day 35, mice in the CF and MF groups received fluoxetine once a day at a dose of 20 mg kg^−1^ by oral administration, while CV and MV mice were treated with the same volume of saline solution.

### Behavior tests

Mice were transferred to the experimentation room for acclimation at least 1 h prior to behavior tests. All tests were conducted in a soundproof room between 10:00 and 18:00. After each test, mice were returned to their home cages and then to the holding room. The body weight of each mouse was measured every week. The OFT, TST, and FST were conducted in the morning with 24 h gap between tests.

#### Open field test

The OF arena consisting of an opaque plastic box (30 × 30 × 30 cm) was thoroughly cleaned with 70% ethanol between tests. On day 36, mice were placed in the center of the open field and allowed to explore for 5 min under dim light. A video tracking system (NeuroVision, Busan, Korea) was used to record the percentage of entries into the center as a measure of psychomotor activity.

#### Tail suspension test

The TST was performed on day 37 according to the method described by Steru *et al*.^60^ with modifications. In brief, a mouse was suspended by its tail from a metal rod using adhesive tape. The rod was fixed 45 cm above the surface of a table in a sound-isolated room. Mice were at least 15 cm from each other, and a styrofoam divider was placed between them. After the six-min test session, the immobility time during the final 5-min of the test was measured using the video tracking system (EthoVision). Mice were considered immobile only when they hung passively and were completely motionless.

#### Forced swim test

On day 38, the FST was performed as previously described^6,61^. Briefly, mice were individually placed in a glass cylinder (20 cm in height × 14 cm in diameter) filled with 16 cm of water (25 ± 1 °C). A styrofoam divider separated the cylinders so that the mice could not see each other during tests. After six min of the swimming test session, immobility time during the six-min interval of the test was measured using the video tracking system (EthoVision, Noldus, Wageningen, Netherlands). Immobility time was measured as the time a mouse stopped struggling and used minimum limb movement to keep its head above the water surface.

### Sample collection and preparation for UHPLC-Q-TOF-MS analysis

#### Hippocampus samples

On day 39 that was 24 h after the final drug or saline administration, mice were sacrificed by decapitation, and the whole brain was removed right away. The hippocampus was carefully separated from the brain on ice. After weighing, the hippocampus was rapidly frozen using liquid nitrogen, and stored at −80 °C until analysis. The whole process was completed within less than 5 min.

A total of 20 mg of hippocampus was extracted in 950 µL of methanol containing 5 μg mL^−1^ of chlorpropamide as an internal standard (IS) by ultrasonic irradiation for 10 min. Precipitated protein was removed by centrifugation at 12,300 *g* for 10 min. The clear supernatant was divided into two aliquots of 450 µL each for positive (POS) ion mode and negative (NEG) ion mode. Each sample was evaporated to dryness under a gentle stream of pure nitrogen gas at room temperature and was reconstituted using 150 µL of methanol. The mixture was passed through a 0.2 µm filter prior to injection into the UHPLC-Q-TOF-MS. Quality control (QC) samples were prepared by mixing the same volume of aliquots from all prepared samples and analyzed every eight samples.

#### Serum samples

Mouse blood was collected in a blood collection tube when the mouse was sacrificed and was allowed to clot for 2 h at 4 °C on ice. The clotting time was recorded. The serum fraction was prepared by centrifugation at 2,500 *g* for 15 min. The supernatant was transferred to a new tube and immediately frozen using liquid nitrogen and stored at −80 °C until analysis.

A total of 150 µL of serum was mixed with 50 µL of 2 μg mL^−1^ of chlorpropamide, and 800 µL of methanol was added to the mixture, followed by thorough mixing on a vortex mixer for 30 s. After protein removal by centrifugation, two aliquots of 400 µL supernatant for each sample were obtained and dried under a stream of pure nitrogen at room temperature. The extract reconstituted in 100 µL of methanol-H_2_O (1:1, v-v) was passed through a 0.2 µm filter prior to injection into the UHPLC-Q-TOF-MS. QC samples were prepared as in 2.5.1.

#### Fecal samples

Mouse feces were collected at weeks 0, 1, 3, and 5. After lyophilization, they were ground to a powder and stored at −20 °C until analysis. Each powder sample weighing 100 mg was spiked with 50 µL of 5 μg mL^−1^ of chlorpropamide and extracted into 950 µL of methanol by thorough mixing on a vortex mixer, followed by sonication for 10 min. After centrifugation at 12,300 *g* for 10 min, the supernatant was directly filtered through a 0.2 µm filter and injected into the UHPLC-Q-TOF-MS. QC samples were prepared as described in 2.5.1.

### Analytical instruments and operating conditions

#### UHPLC conditions

UHPLC analysis was performed using an Acquity UPLC system (Waters Co., Milford, MA, USA) equipped with a binary solvent delivery system, a cooling autosampler (maintained at 4 °C), and a thermostatically controlled column compartment. All samples were analyzed in both POS and NEG ion modes. The flow rate and injection volume were 0.35 mL min^−1^ and 5 µL, respectively, for all sample types.

Hippocampus samples were chromatographed on a ZORBAX Rapid Resolution High Definition Eclipse Plus C18 column (100 mm × 2.1 mm, 1.8 µm) from Agilent (Santa Clara, CA, USA), which was maintained at 45 °C. The mobile phase was composed of solvent A, 10 mM ammonium formate +0.1% formic acid (POS) or 0.1% formic acid (NEG), and solvent B, acetonitrile containing 0.1% formic acid. A solvent gradient system was used as follows: 0–2 min, 10–85% B; 2–8 min, 85–90% B; 16–21 min, 100% B. Between runs, the system was allowed to equilibrate at the initial conditions for an additional 3 min.

Serum samples were analyzed under the same conditions as used for the hippocampus samples except for the column temperature, mobile phase, and gradient program. The column temperature was maintained at 30 °C, and 0.1% formic acid (solvent A) and acetonitrile containing 0.1% formic acid (solvent B) were used for both POS and NEG ion modes. The elution gradient program was: 0–2 min, 5–25% B; 2–17 min, 25–70% B; 20–21 min, 100% B.

Fecal samples were chromatographed on an Acquity UHPLC BEH C18 column (50 mm × 2.1 mm, 1.7 µm) from Waters (Milford, MA, USA) maintained at 30 °C. A gradient elution (0–1 min, 1–20% B; 1–15 min, 20–60% B; 15–20 min, 60–100% B; 20–21 min, 100% B) was performed using the same mobile phase as used for the serum samples.

#### MS conditions

MS analysis was conducted using a Waters Acquity Xevo G2 Q-TOF tandem mass spectrometer (Waters Corp., Manchester, UK) equipped with an electrospray ionization (ESI) interface. Instrument parameters were set as follows: capillary voltage, 3.0 kV (POS)/2.0 kV (NEG); sample cone, 30 V (POS)/45 V (NEG); extraction cone, 4.0 V; source temperature, 120 °C; desolvation temperature, 300 °C; desolvation gas (nitrogen), 600 L h^−1^. The instrument was controlled by Masslynx software (version 4.1, Waters Corp., Milford, MA, USA), which was also used for raw data acquisition and processing. Mass calibration was performed by direct infusion of 5 mM sodium formate solution. Data were acquired from *m/z* 50 to 1500 Da and corrected during acquisition using a lock spray composed of 2 μg mL^−1^ leucine enkephalin (*m/z* 556.2771 for POS and 554.2615 for NEG) solution infused at a flow rate of 20 μL min^−1^. The high collision energy ramp ranged from 20 to 45 V.

### Statistical data analysis

Data from the behavioral tests were expressed as mean ± standard error of the mean (SEM). Statistical analysis was performed using one-way ANOVA test and two-way ANOVA test, which were followed by Tukey’s multiple comparison test and Fisher’s LSD test, respectively, for post-hoc analysis using Prism 6.0 (GraphPad Software, Inc. USA). Significant differences were indicated at levels of *p* < 0.05, *p* < 0.01, and *p* < 0.001.

For metabolic profiles, the raw data were analyzed using MarkerLynx Applications Manager (version 4.1), which allowed deconvolution, alignment, and data reduction to give a list of mass and retention time pairs with corresponding intensities for all detected peaks from each data file in the dataset. The parameters were set as follows: RT window, 0.05 min; mass window, 0.05 Da; noise elimination level, three standard deviations above background; and intensity threshold, 20 counts per second. The resulting data were analyzed by EZinfo software using multivariate statistical analysis methods including principal component analysis (PCA) and pair-wise orthogonal projections to latent structures discriminant analysis (OPLS-DA).

### Metabolite identification

Identification of the differential low molecular weight metabolites was performed based on the metabolomics database using Metlin (http://metlin.scripps.edu/index.php), the Mouse Multiple Tissue Metabolome Database (http://mmdb.iab.keio.ac.jp/), and The Human Metabolome Database (http://www.hmdb.ca/). Specifically, accurate *m/z* values of molecular ions were put into relevant online for preliminary identification, and then, the MS/MS fragments obtained from 20–45 V collision energy were compared with those in Metlin. Finally, potential marker metabolites were identified by comparison of acquired parent ions and fragment ions with those of commercially available standards and/or the database.

## Supplementary information

Supplementary information

## Data Availability

The data in this study may be available from the corresponding author upon request.
