# Supplementary material for: A comprehensive metabolomics investigation of hippocampus, serum, and feces affected by chronic fluoxetine treatment using the chronic unpredictable mild stress mouse model of depression
Source: Sci Rep. 2019 May 20;9:7566. doi: 10.1038/s41598-019-44052-2 (PMC6527582; doi:10.1038/s41598-019-44052-2)

Supplementary Information

A comprehensive metabolomics investigation of hippocampus, serum, and feces affected by chronic fluoxetine treatment using the chronic unpredictable mild stress mouse model of depression

Jing Zhao^§, ┼^, Yang-Hee Jung^§^, Yan Jin, Seulgi Kang, Choon-Gon Jang, Jeongmi Lee^*^

School of Pharmacy, Sungkyunkwan University, Suwon 16419, Republic of Korea

^§^ These authors equally contributed.

^┼^ Current address: Shenyang Pharmaceutical University, Shenyang 110016, PR China

^*^ Corresponding author: jlee0610@skku.edu.

Tel.: +82-31-290-7784/ Fax: +82-31-292-8800

**Table S1.** Summary of the parameters for assessing PCA modeling quality

| Model | ESI + | | ESI - | |
| --- | --- | --- | --- | --- |
|  | R^2 a^ | Q^2 b^ | R^2^ | Q^2^ |
| Hippocampus | 0.822 | 0.719 | 0.781 | 0.706 |
| Serum | 0.848 | 0.774 | 0.835 | 0.761 |
| Feces | 0.912 | 0.907 | 0.972 | 0.893 |

^a^ R^2^ are the cumulative modeled variation in the response variables.

^b^ Q^2^ is the cumulative predicted variation of the model.

**Table S2.** Summary of the parameters for assessing OPLS-DA modeling quality

| Sample | Group | ESI + | | | ESI - | | |
| --- | --- | --- | --- | --- | --- | --- | --- |
|  |  | R^2^X ^a^ | R^2^Y ^a^ | Q^2 a^ | R^2^X | R^2^Y | Q^2^ |
| Hippocampus | CV-MV | 0.731 | 0.988 | 0.921 | 0.711 | 0.971 | 0.892 |
|  | MV-MF | 0.764 | 0.964 | 0.881 | 0.701 | 0.954 | 0.904 |
|  | CV-CF | 0.697 | 0.874 | 0.836 | 0.672 | 0.781 | 0.840 |
| Serum | CV-MV | 0.885 | 0.991 | 0.941 | 0.713 | 0.740 | 0.790 |
|  | MV-MF | 0.910 | 0.911 | 0.989 | 0.898 | 0.941 | 0.884 |
|  | CV-CF | 0.658 | 0.772 | 0.796 | 0.651 | 0.757 | 0.834 |
| Feces | CV-MV | 0.844 | 0.986 | 0.911 | 0.901 | 0.932 | 0.845 |
|  | MV-MF | 0.833 | 0.931 | 0.945 | 0.721 | 0.781 | 0.799 |
|  | CV-CF | 0.791 | 0.923 | 0.883 | 0.689 | 0.751 | 0.821 |

^a^ R^2^X and R^2^Y are the cumulative modeled variation in X and Y matrix, respectively. Q^2^ is the cumulative predicted variation.

**Fig. S1.** PCA score plots of samples prepared from hippocampus (a, b), serum (c, d), and feces (e, f). (a, c, and e obtained in POS ion mode; b, d, and f obtained in NEG ion mode).

(a)
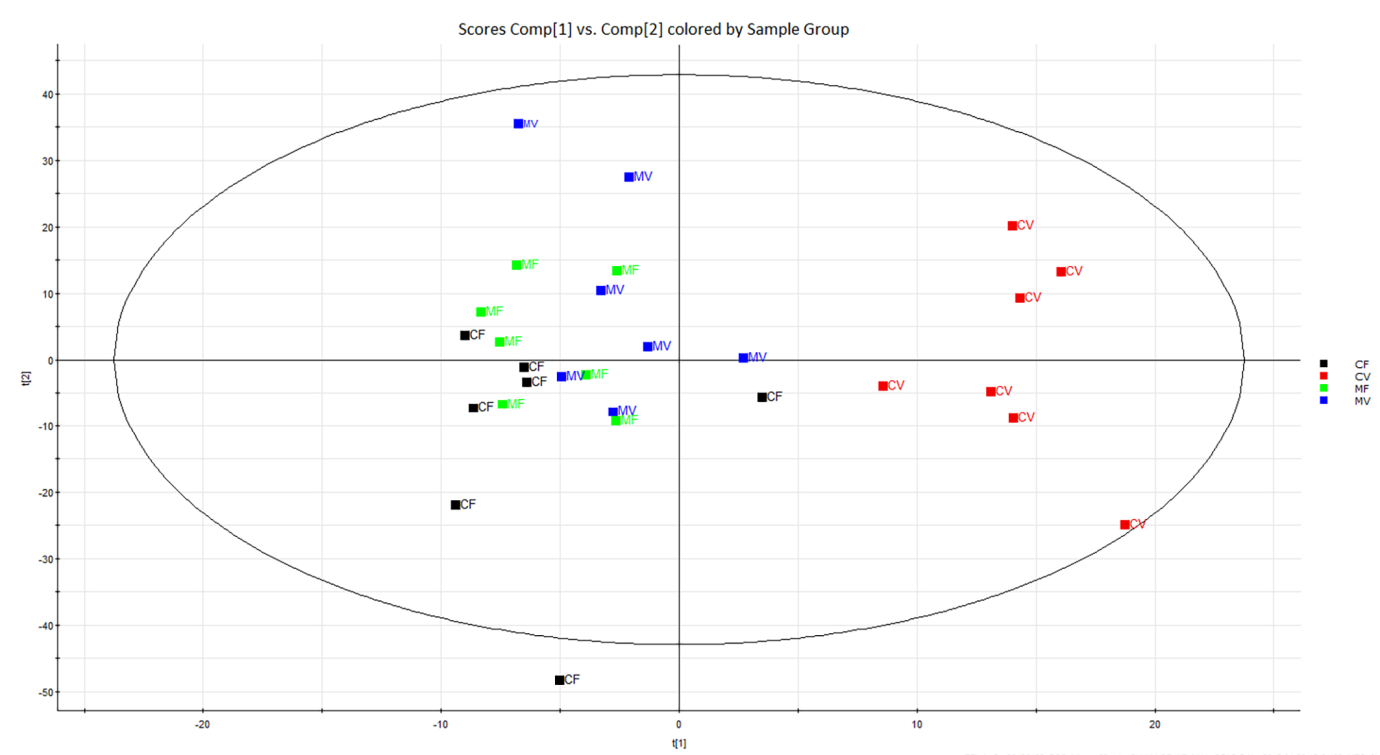


(b)
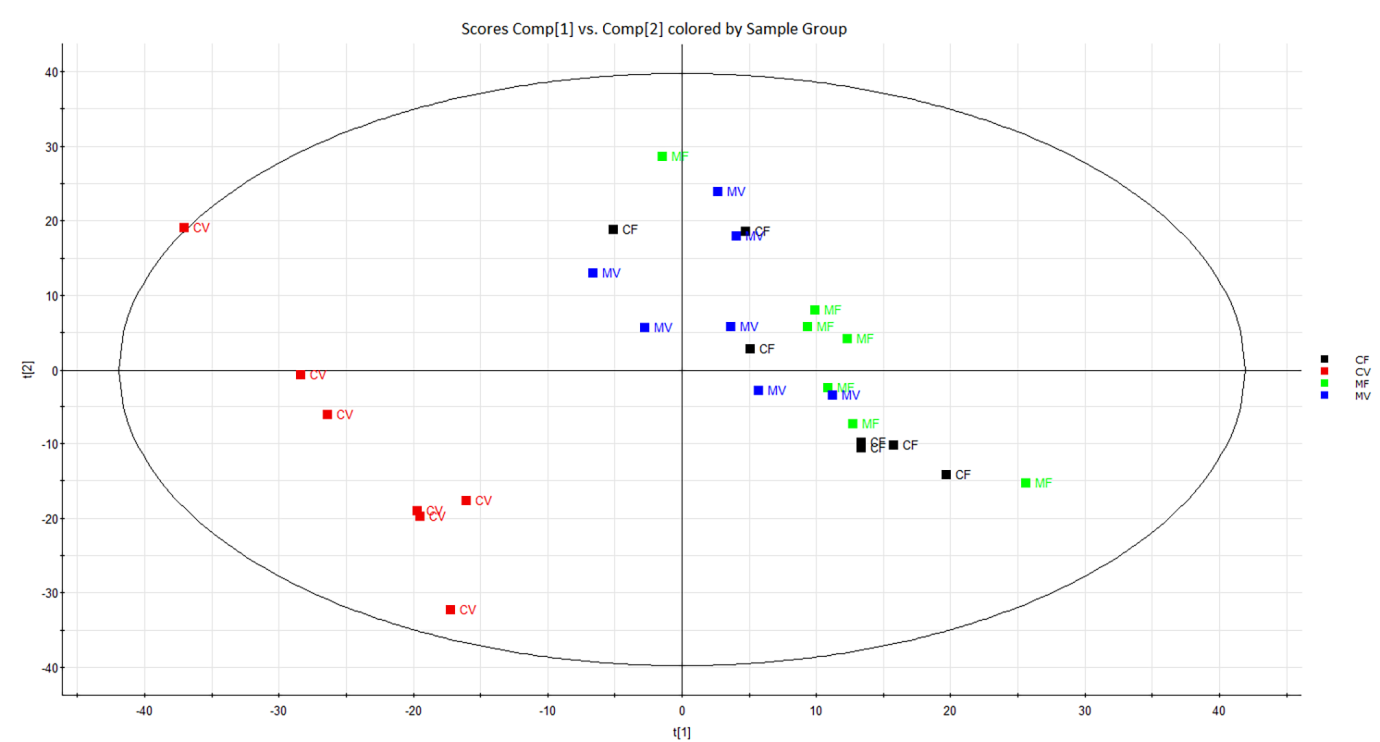


(c)
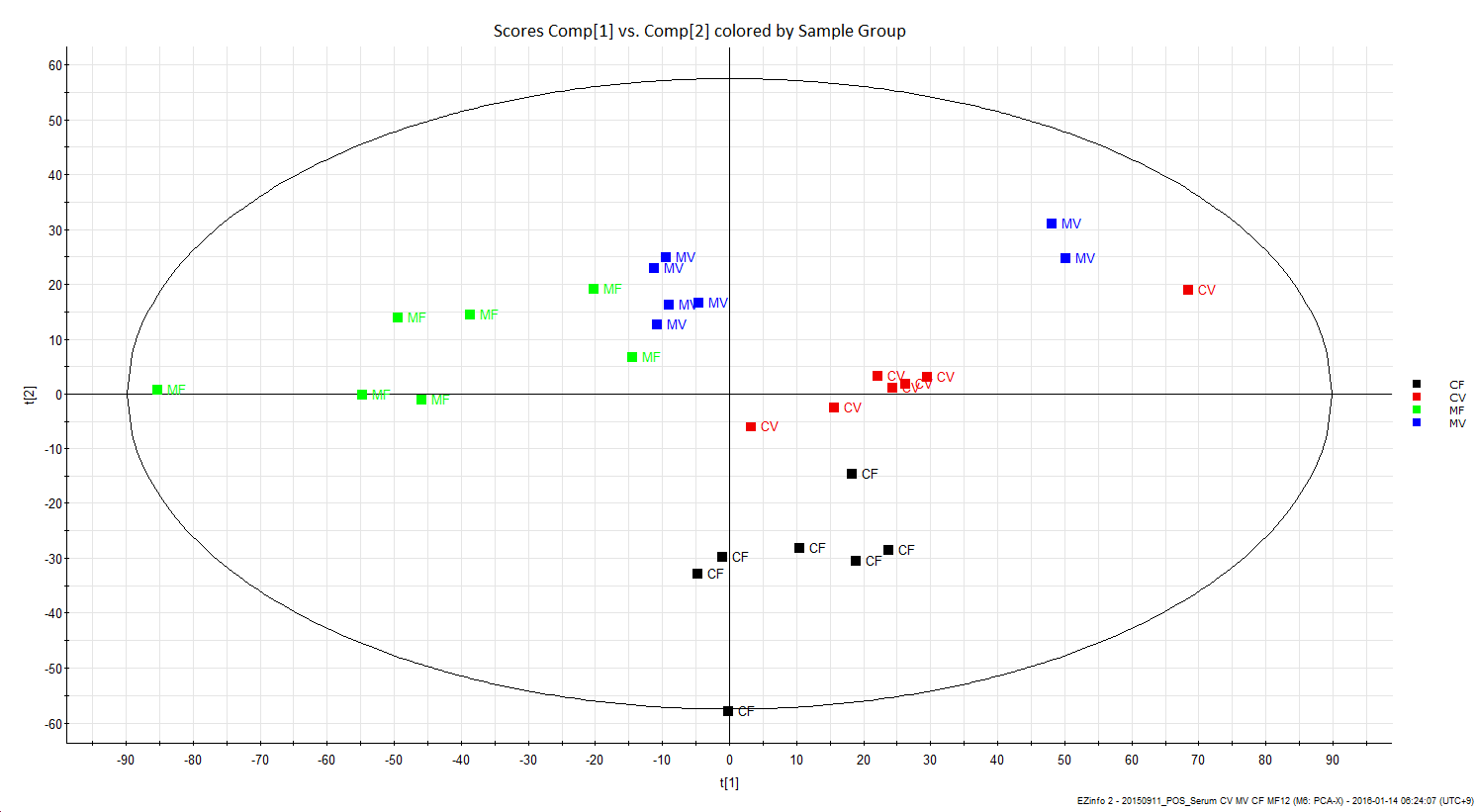


(d)
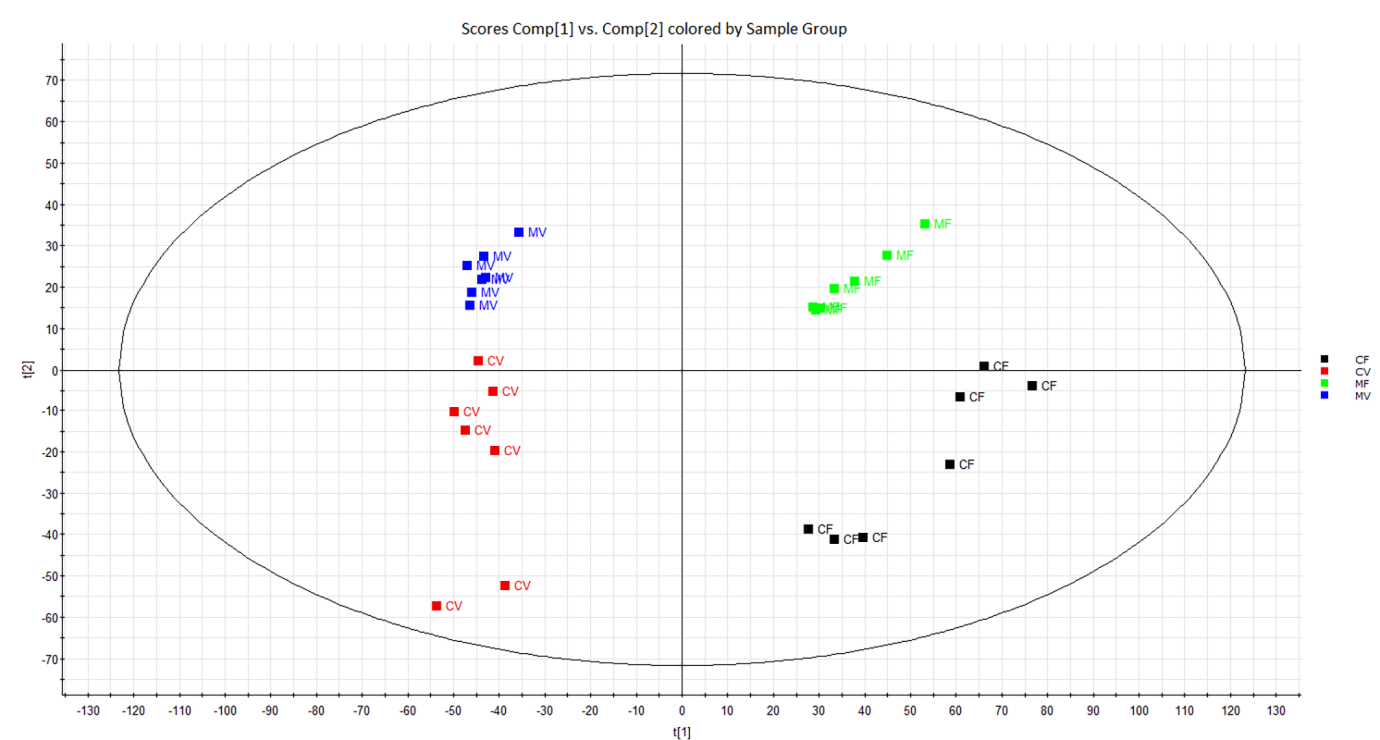


(e)
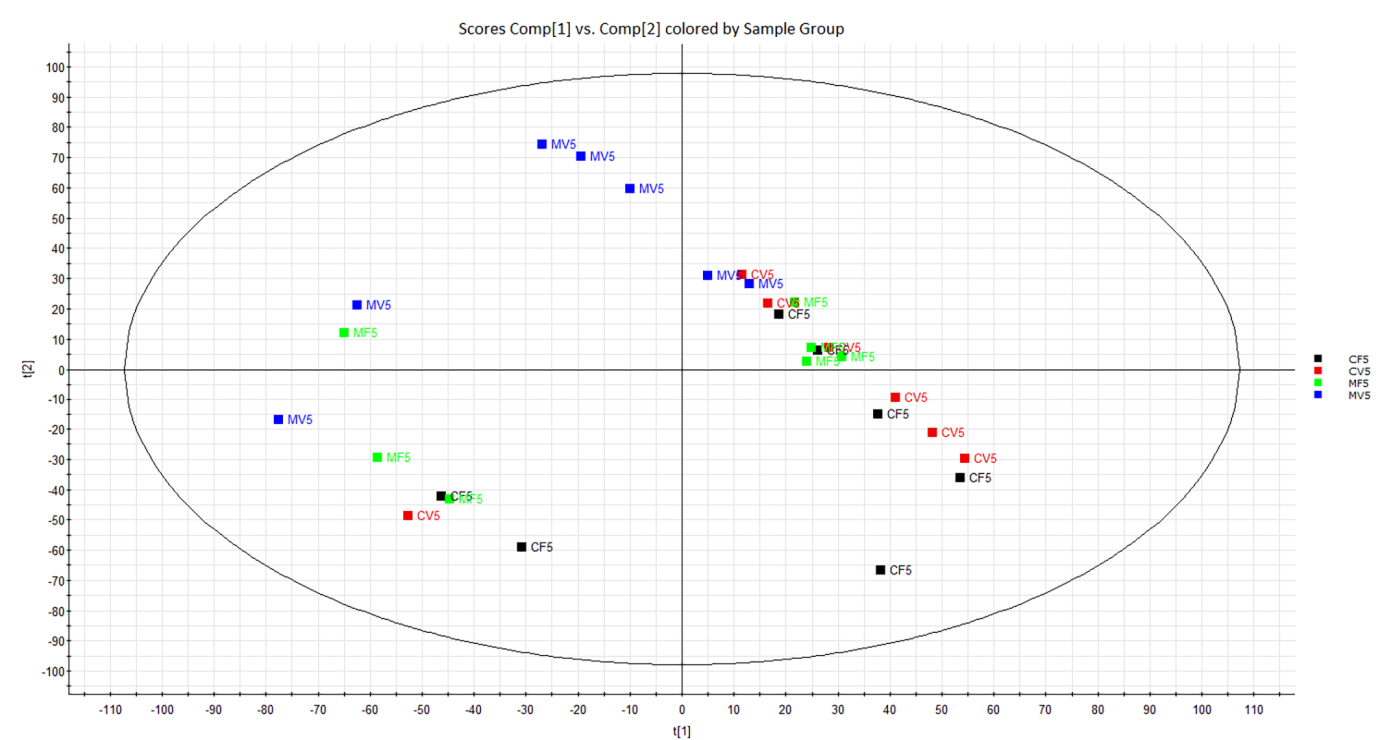


(f)
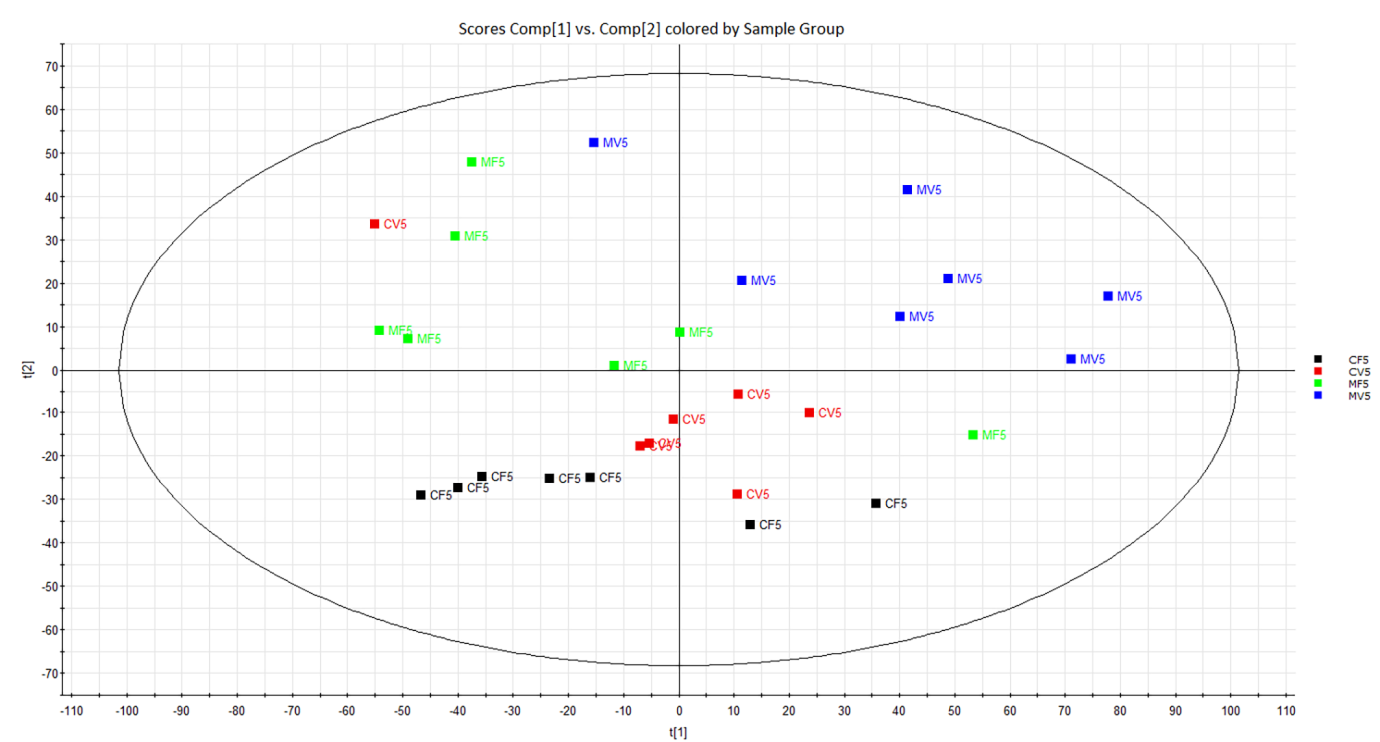


**Fig. S2.** PCA score plots of samples prepared from hippocampus (a, b), serum (c, d), and feces (e, f) including QC samples. (a, c, and e obtained in POS ion mode; b, d, and f obtained in NEG ion mode).

(a)
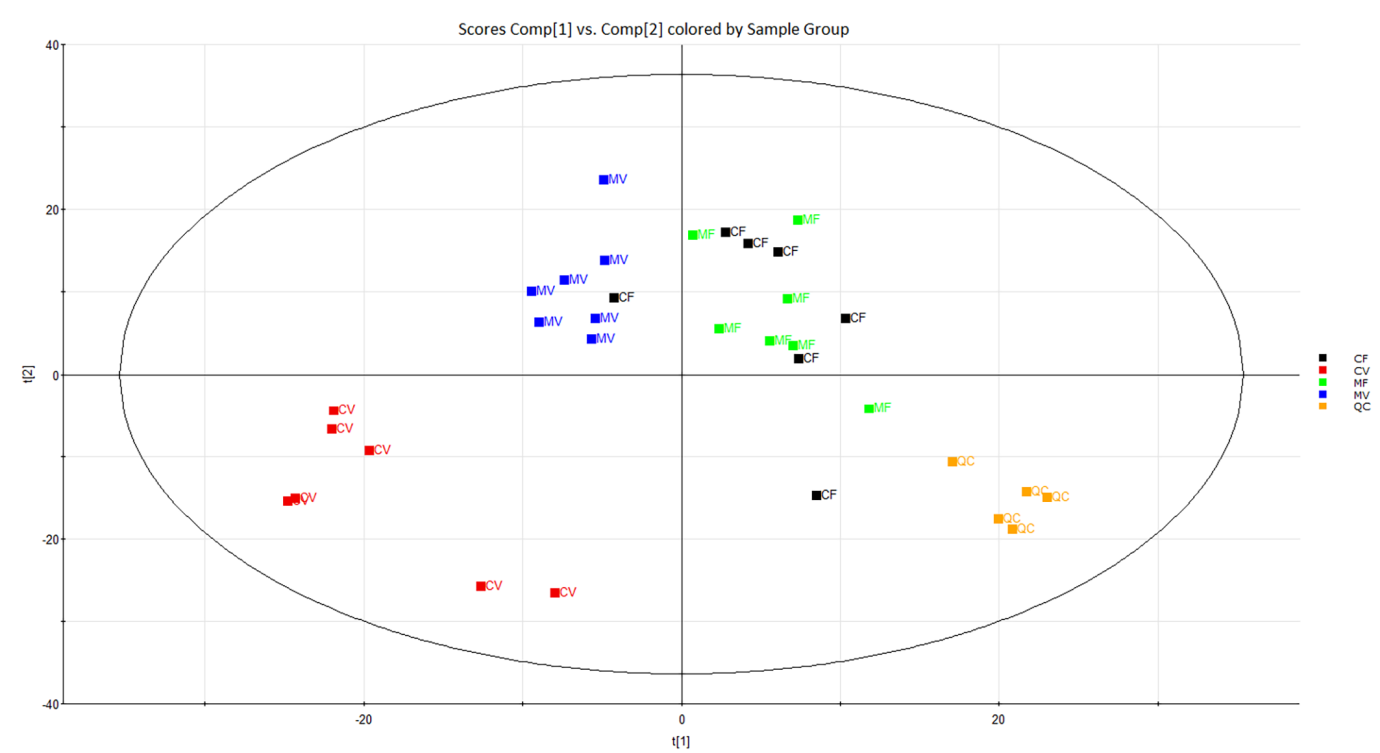


(b)
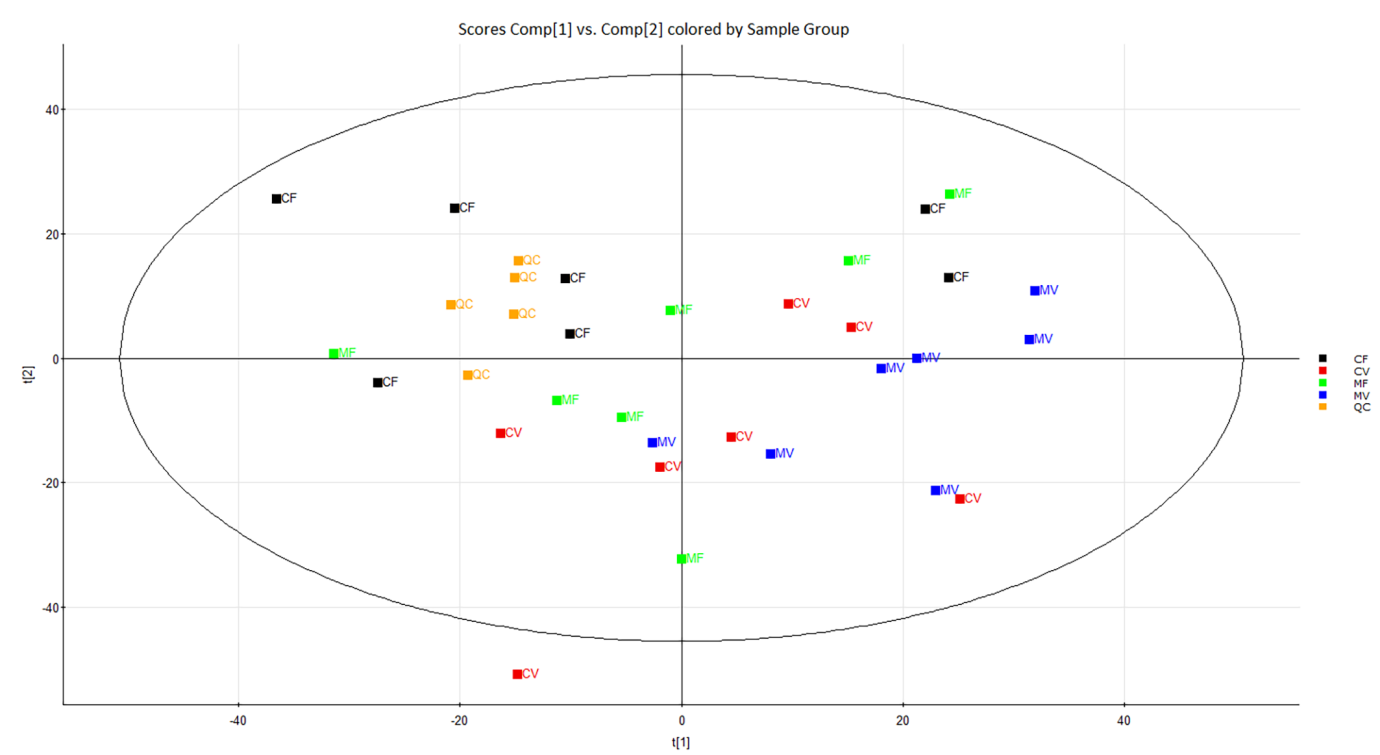


(c)

**
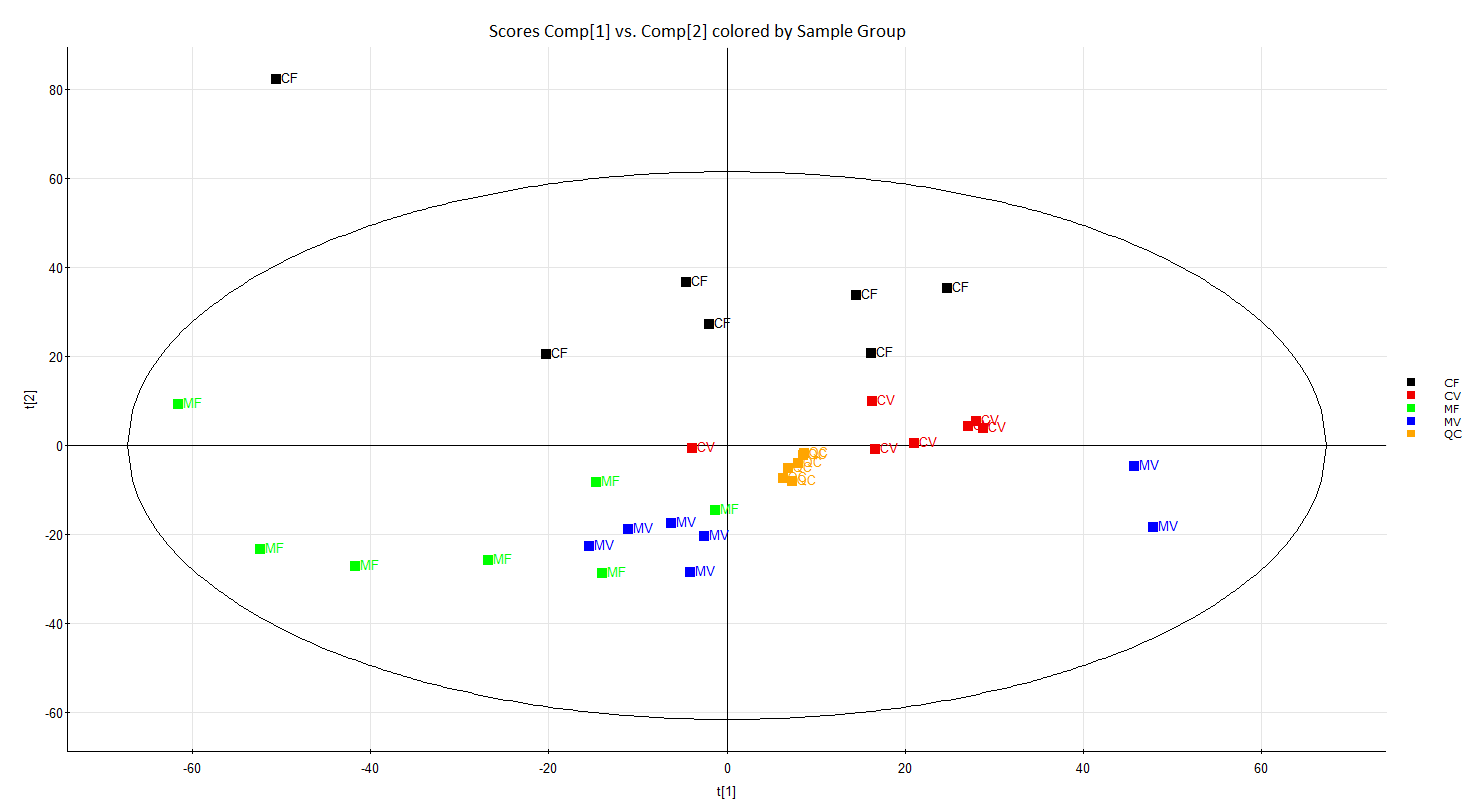
**

(d)

**
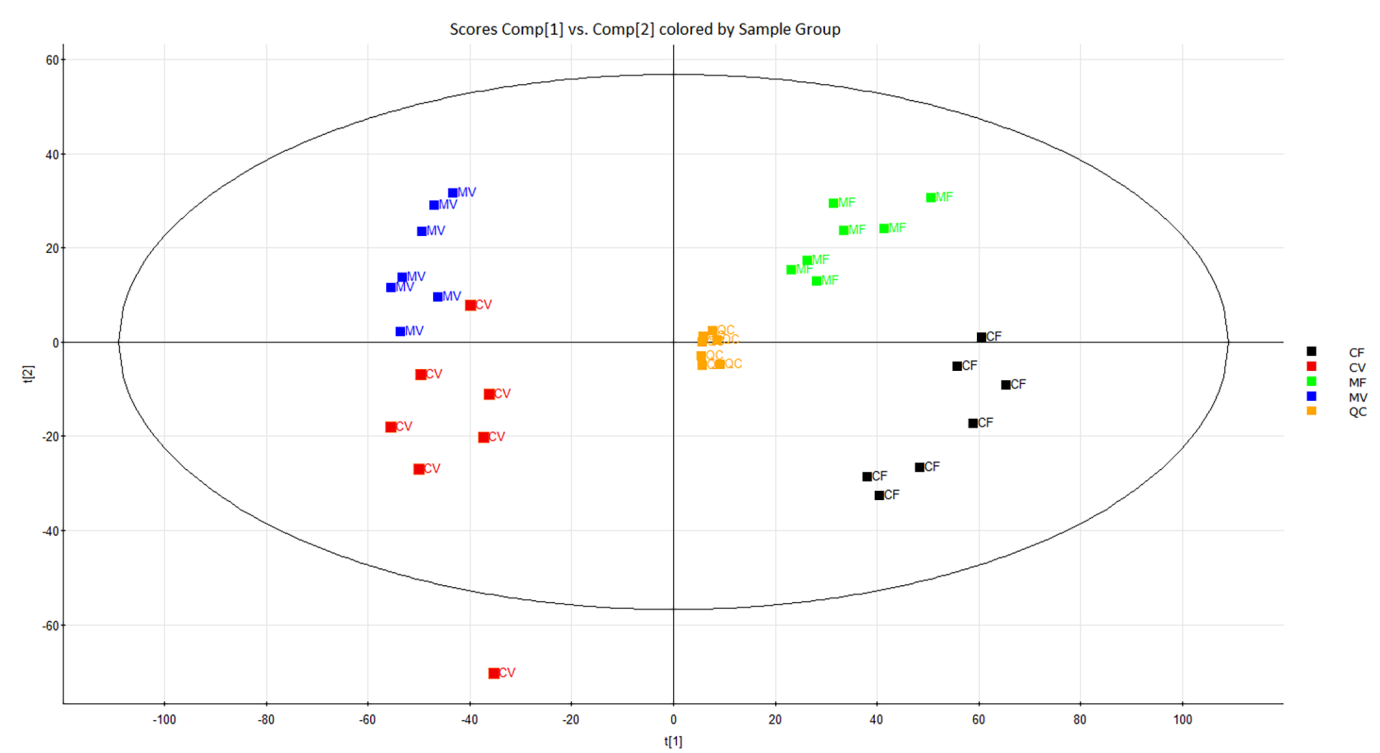
**

(e)

**
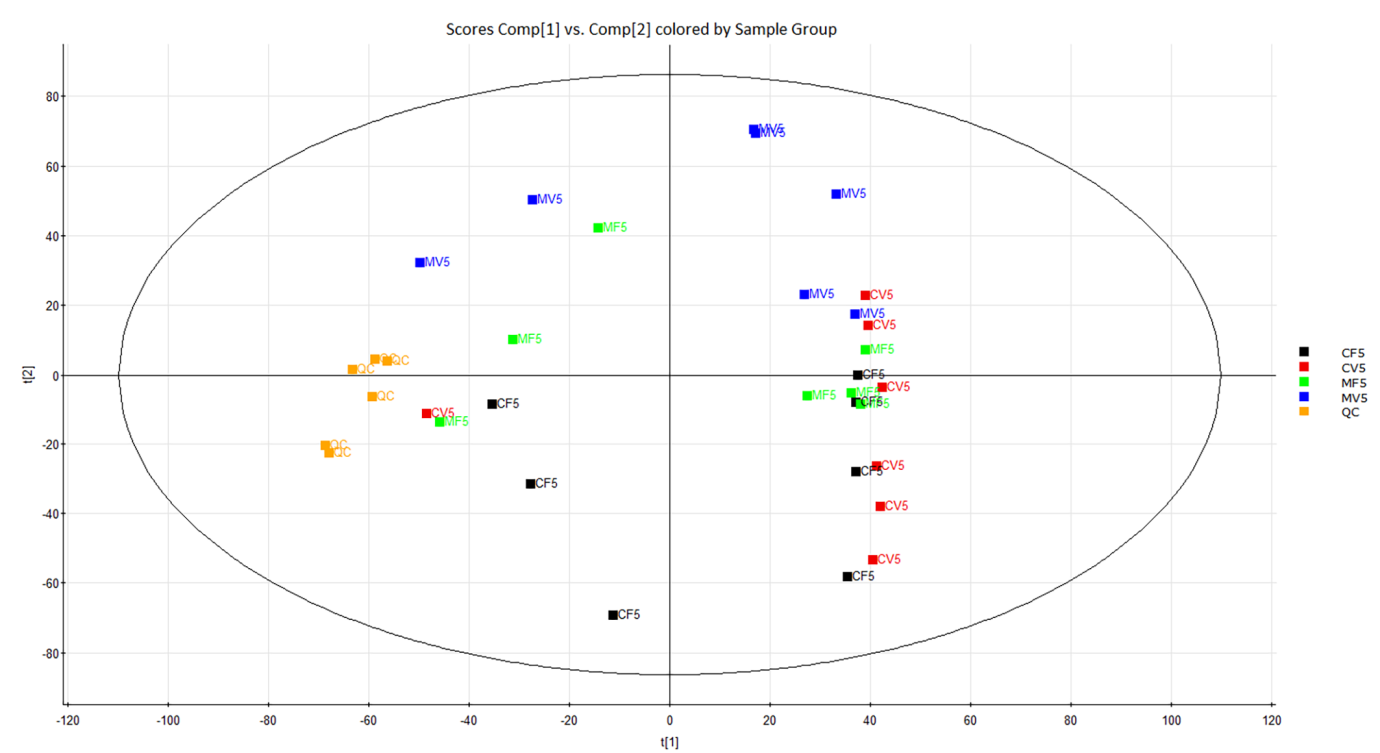
**

(f)

**
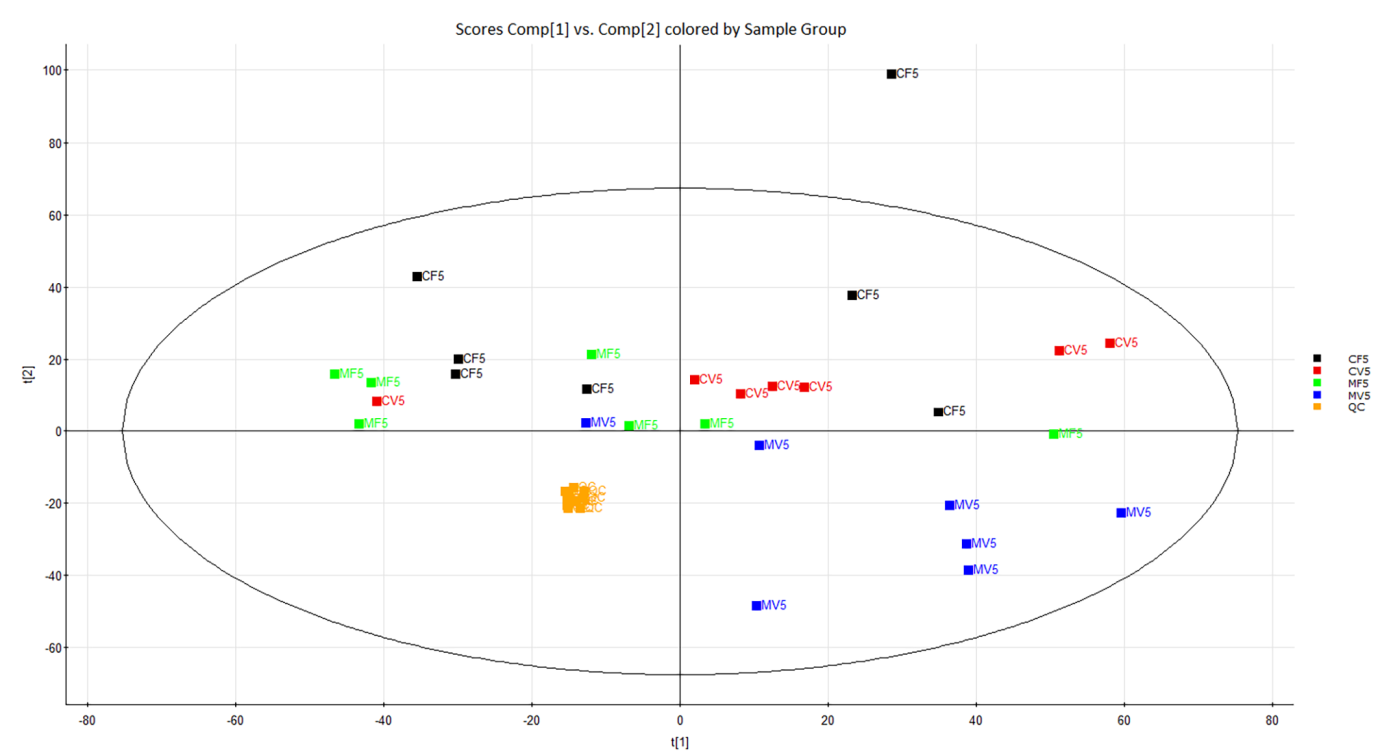
**

**Fig. S3.** OPLS-DA score plots for pair-wise comparisons between CV and MV (a, b), MV and MF (c, d), and CV and CF groups (e, f) from hippocampus samples. (a, c, e in POS ion mode; b, d, f in NEG ion mode)

(a)
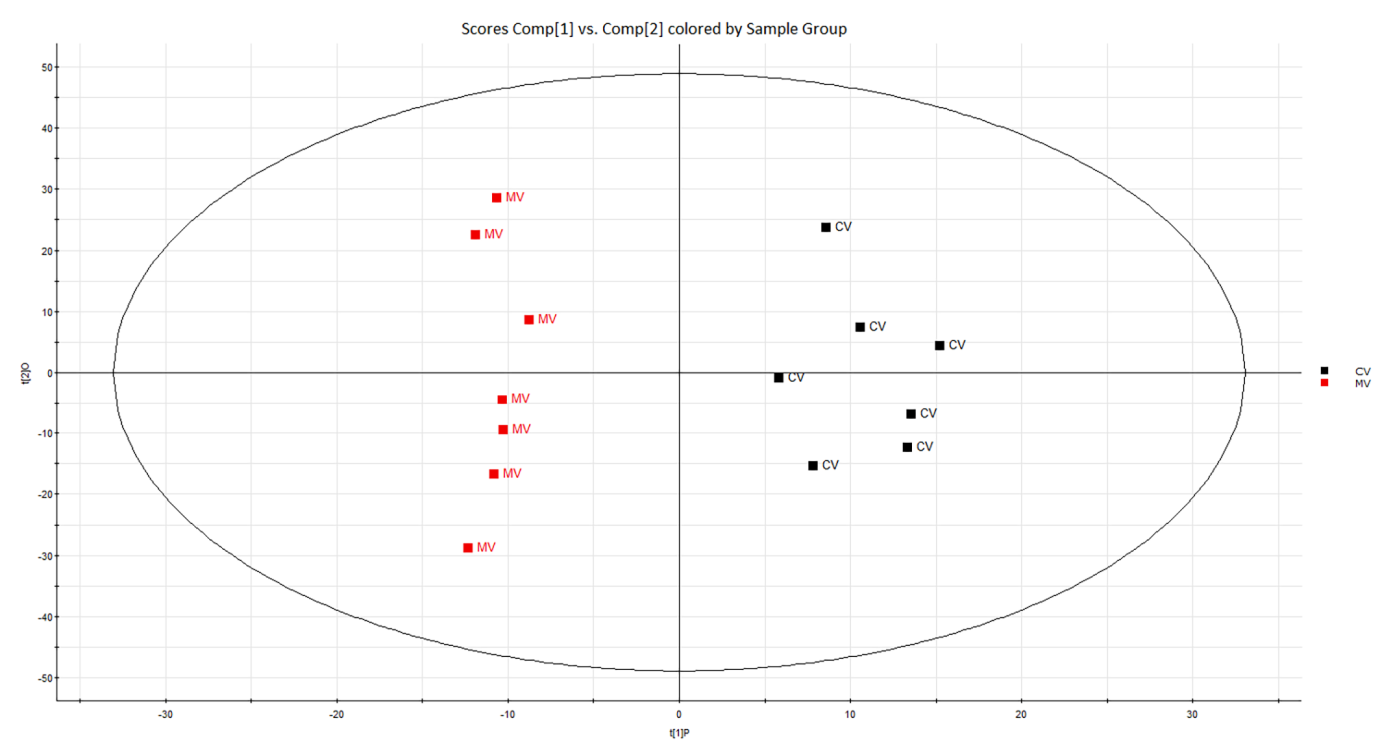


(b)
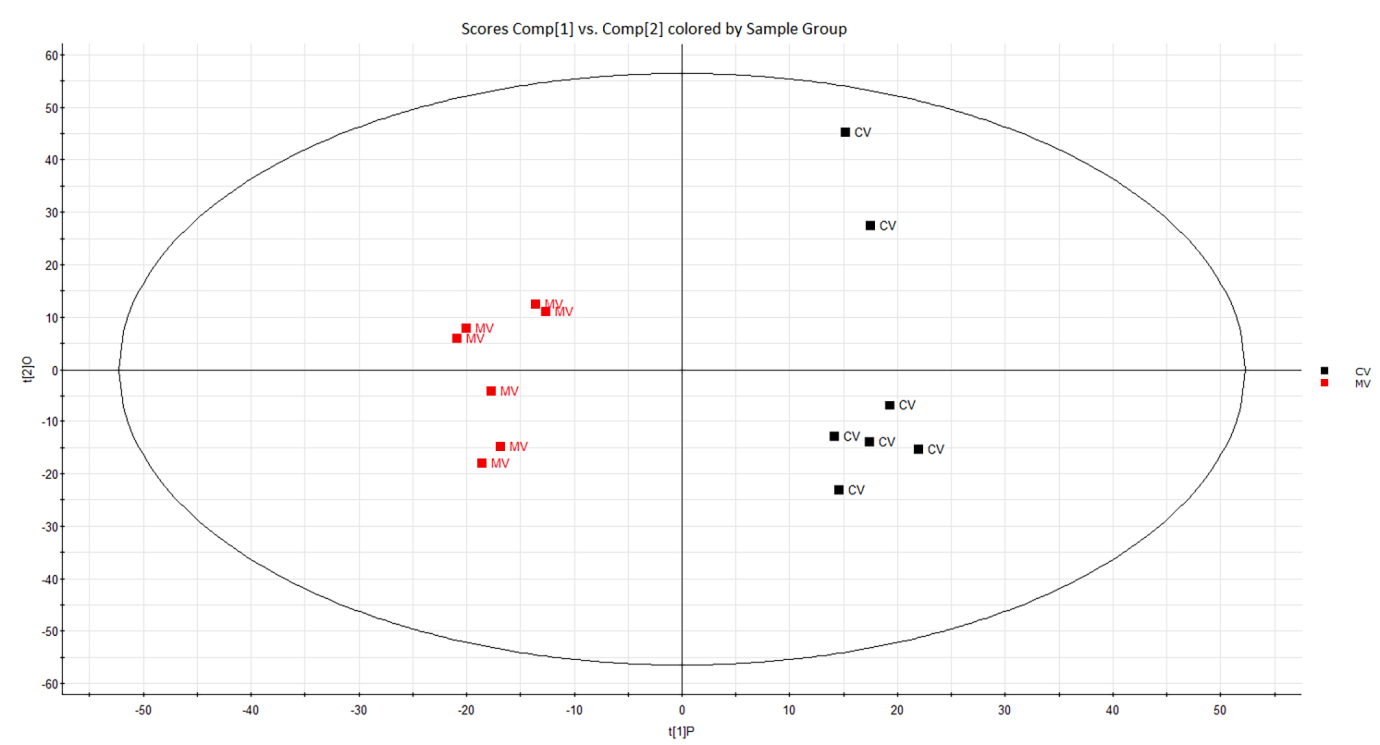


(c)
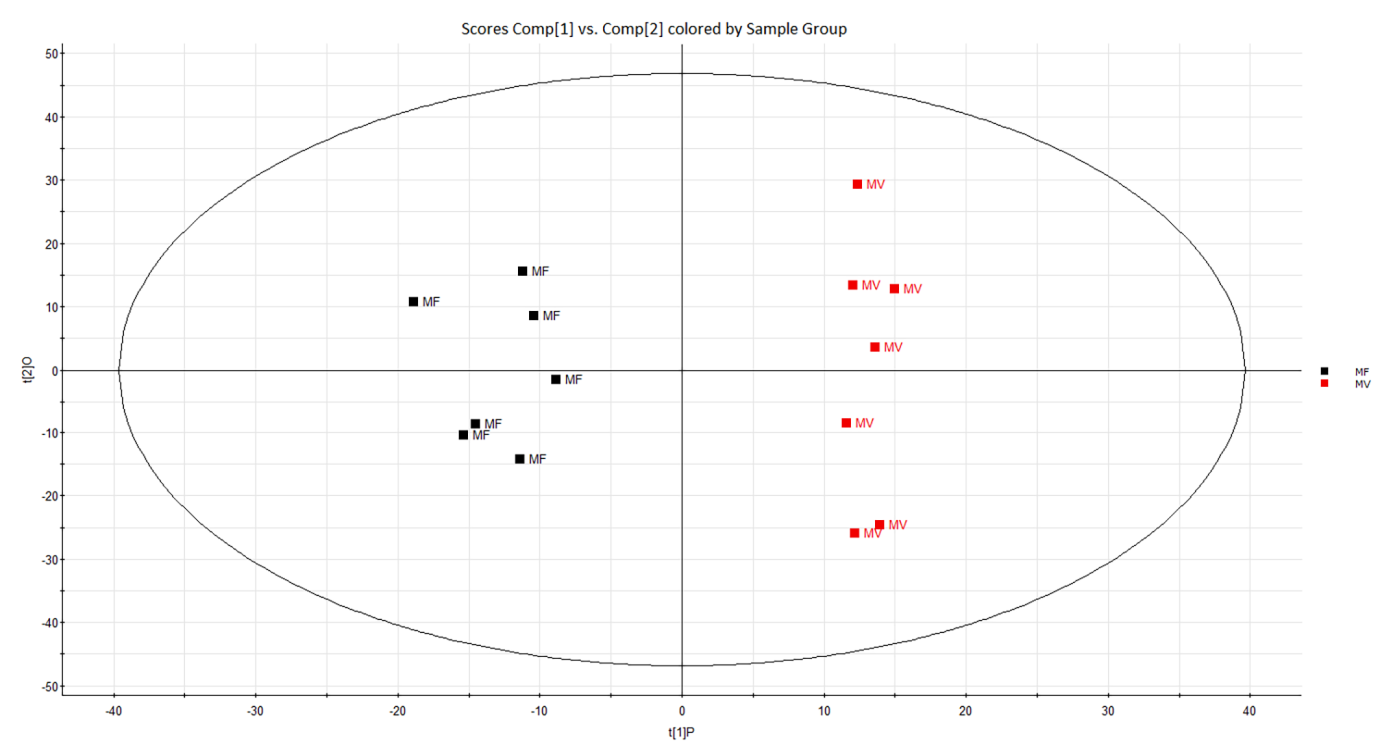


(d)
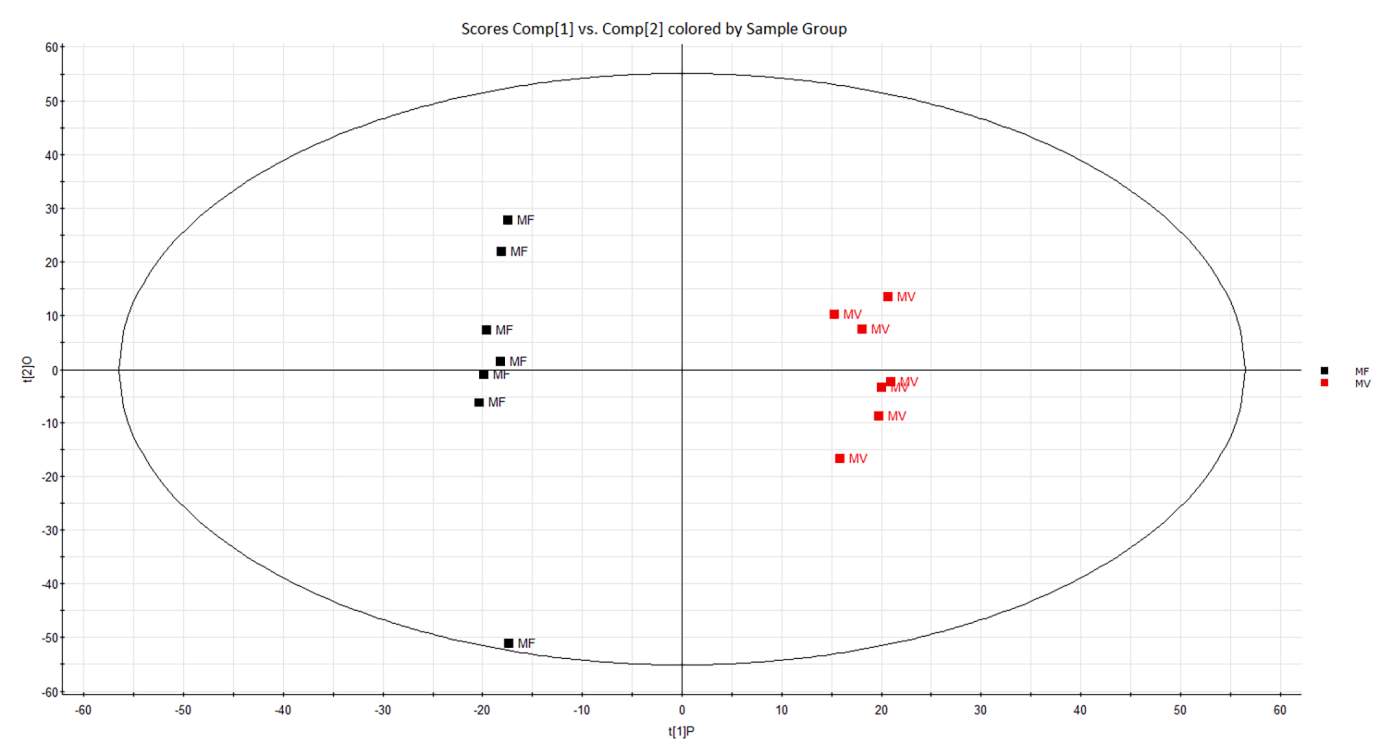


(e)
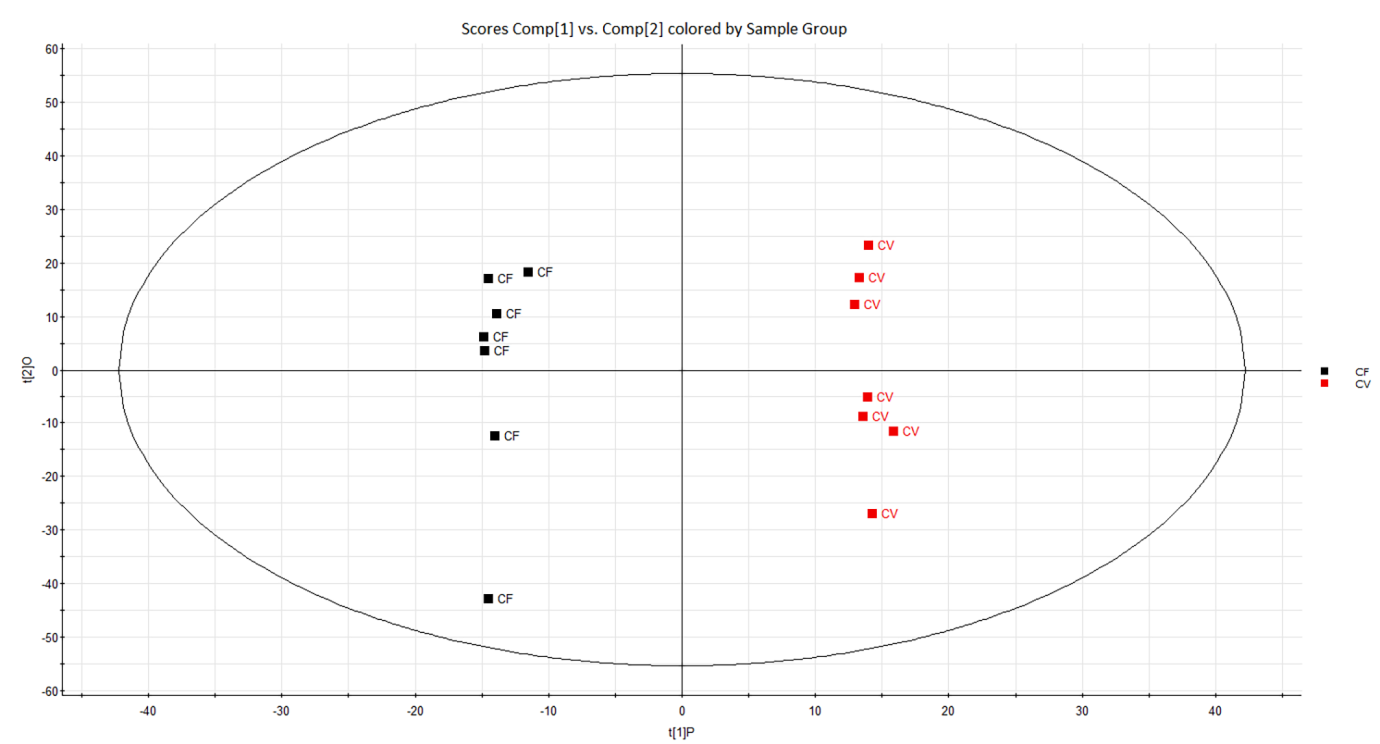


(f)
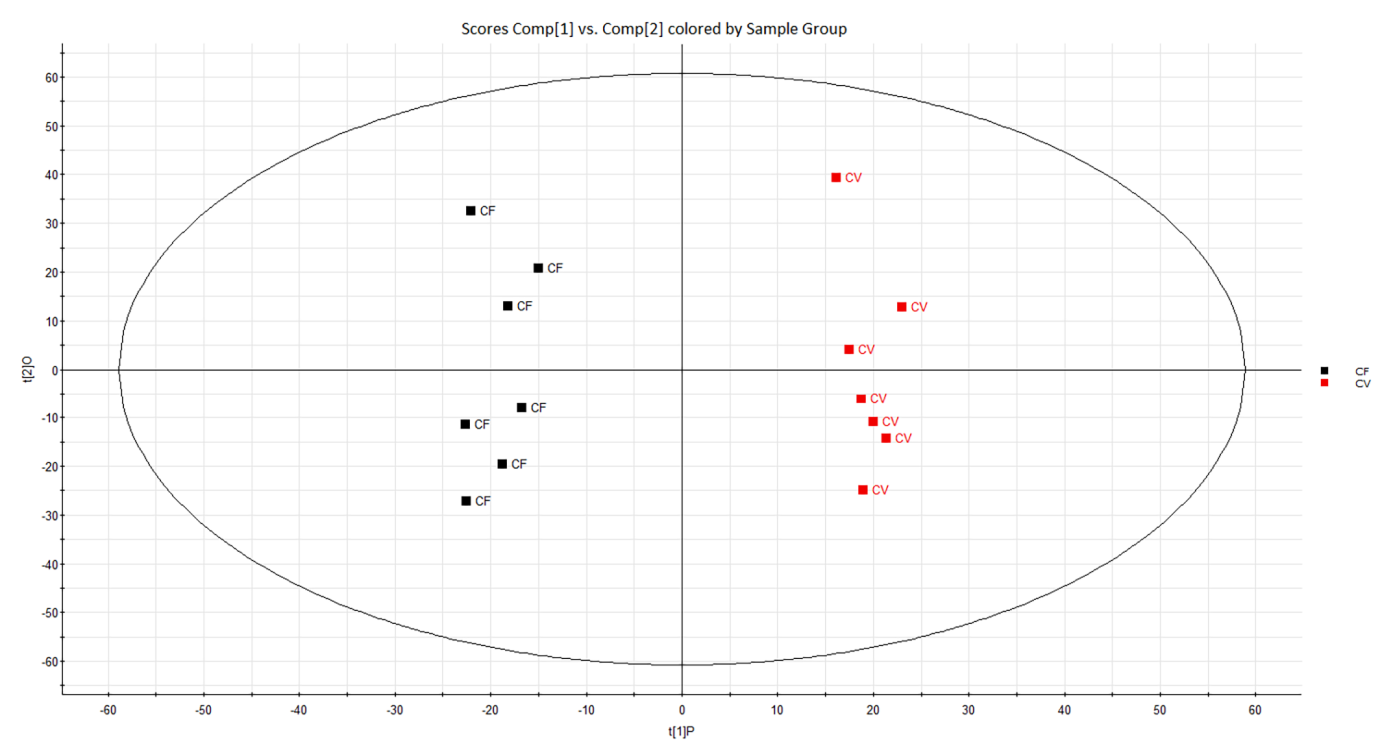


**Fig. S4.** OPLS-DA score plots for pair-wise comparisons between CV and MV (a, b), MV and MF (c, d), and CV and CF groups (e, f) from serum samples. (a, c, e in POS ion mode; b, d, f in NEG ion mode)

(a)
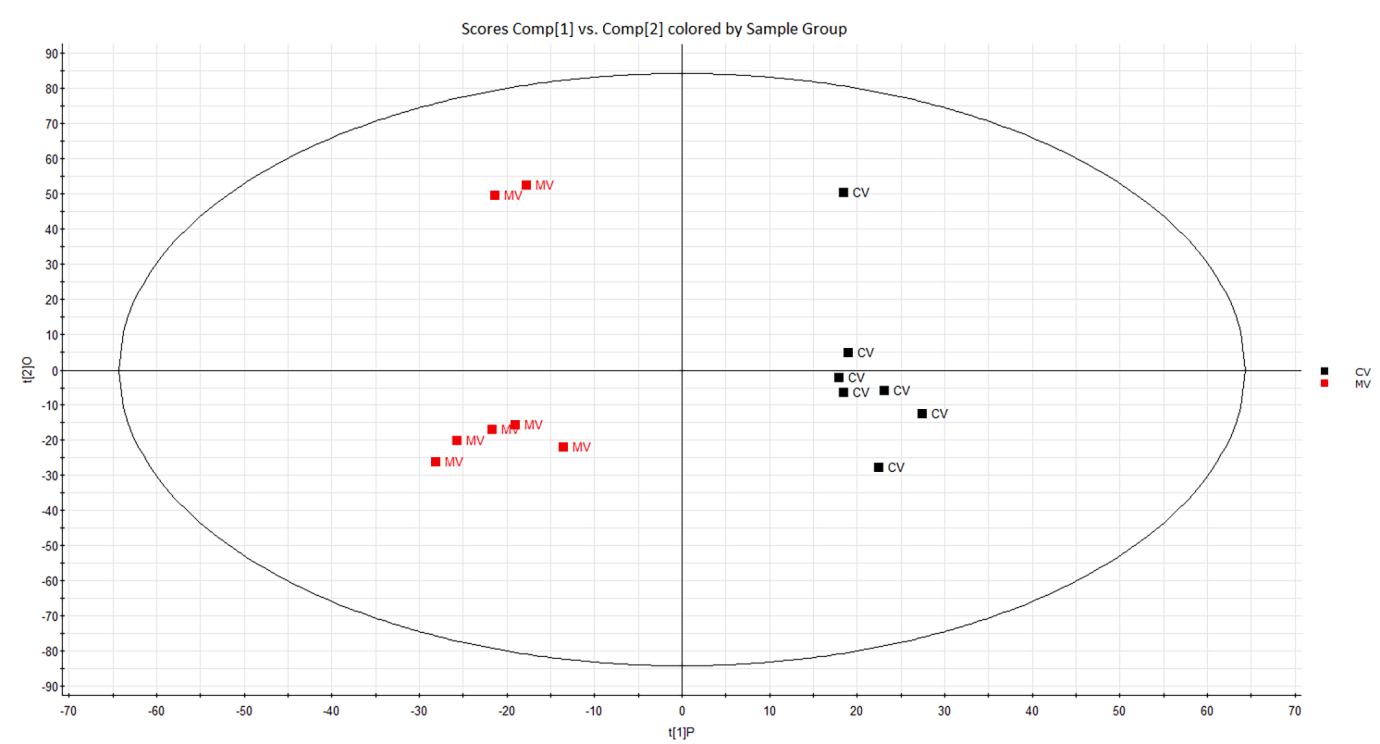


(b)
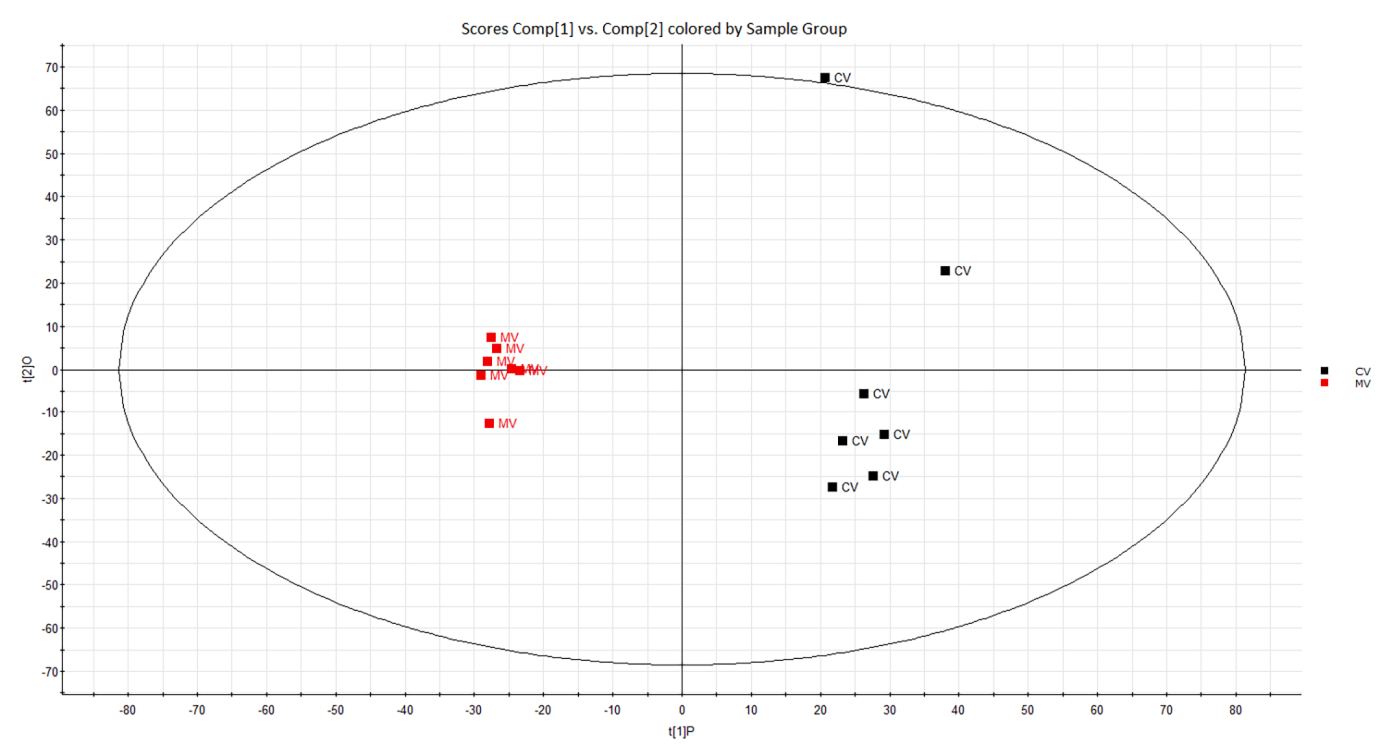


(c)
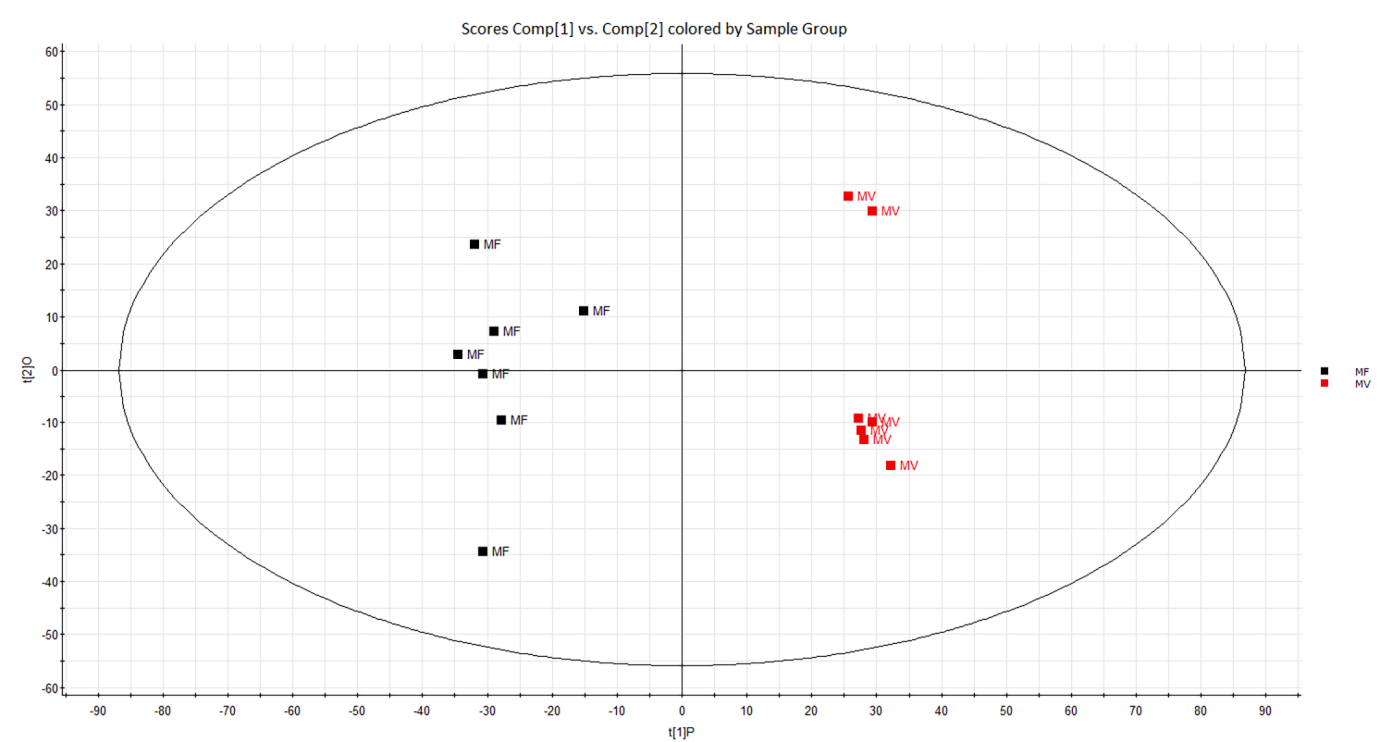


(d)
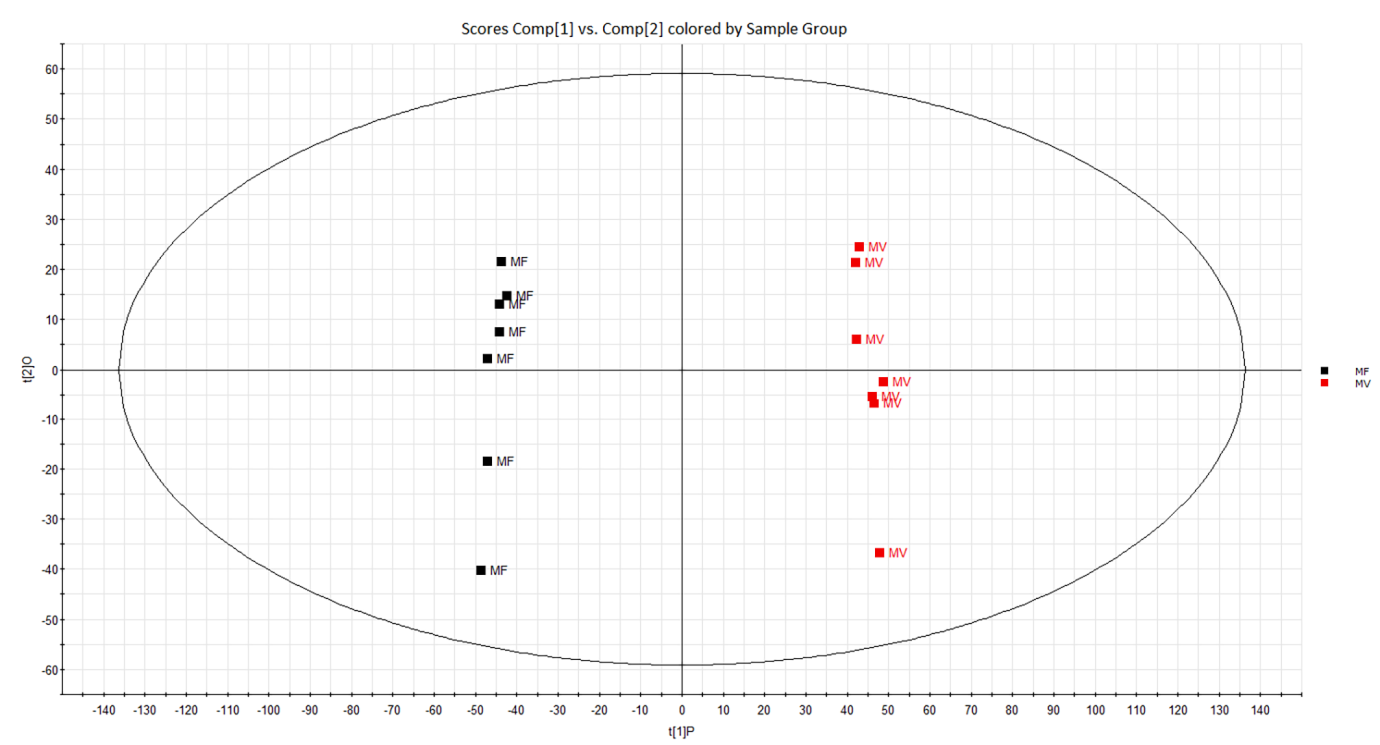


(e)
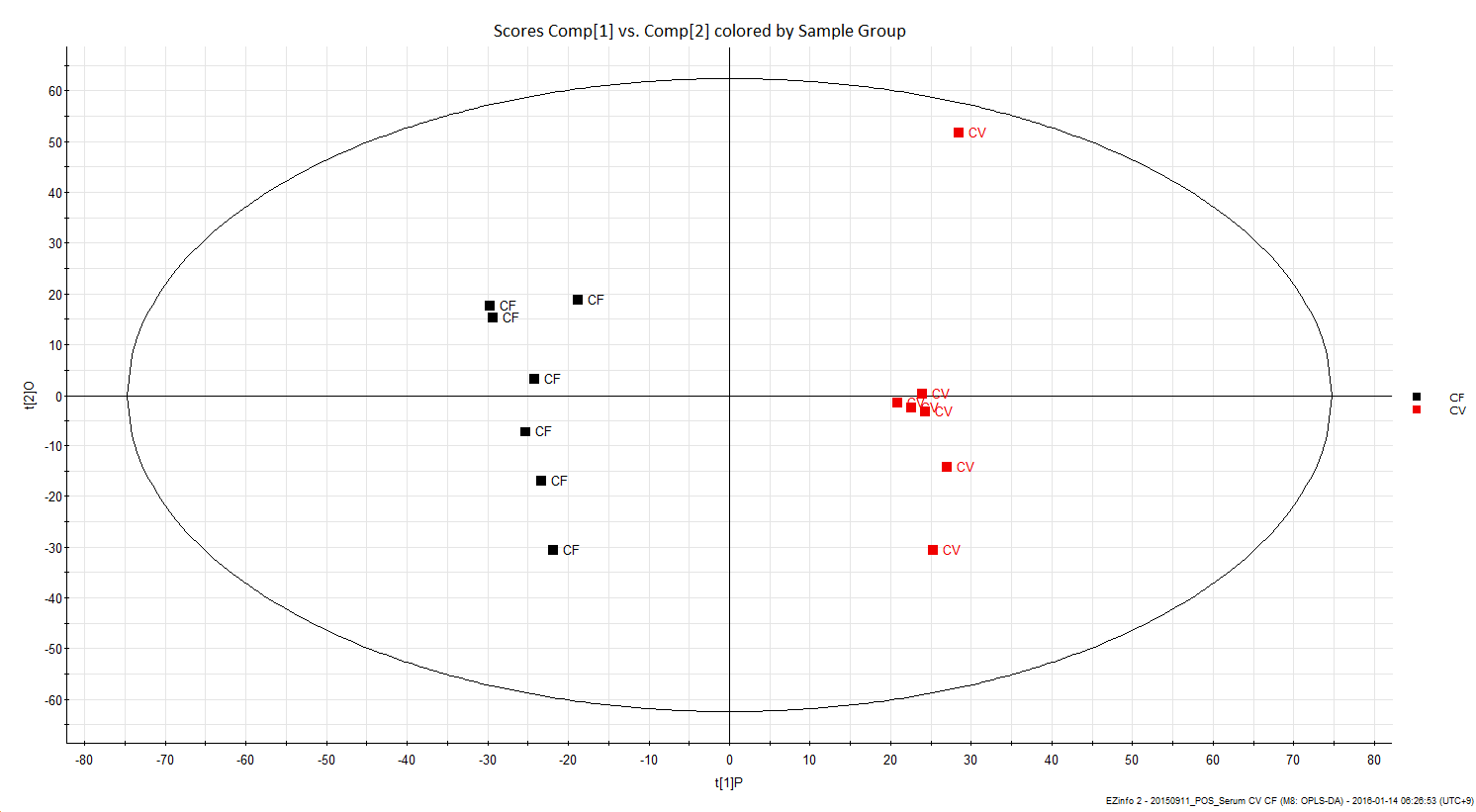


(f)
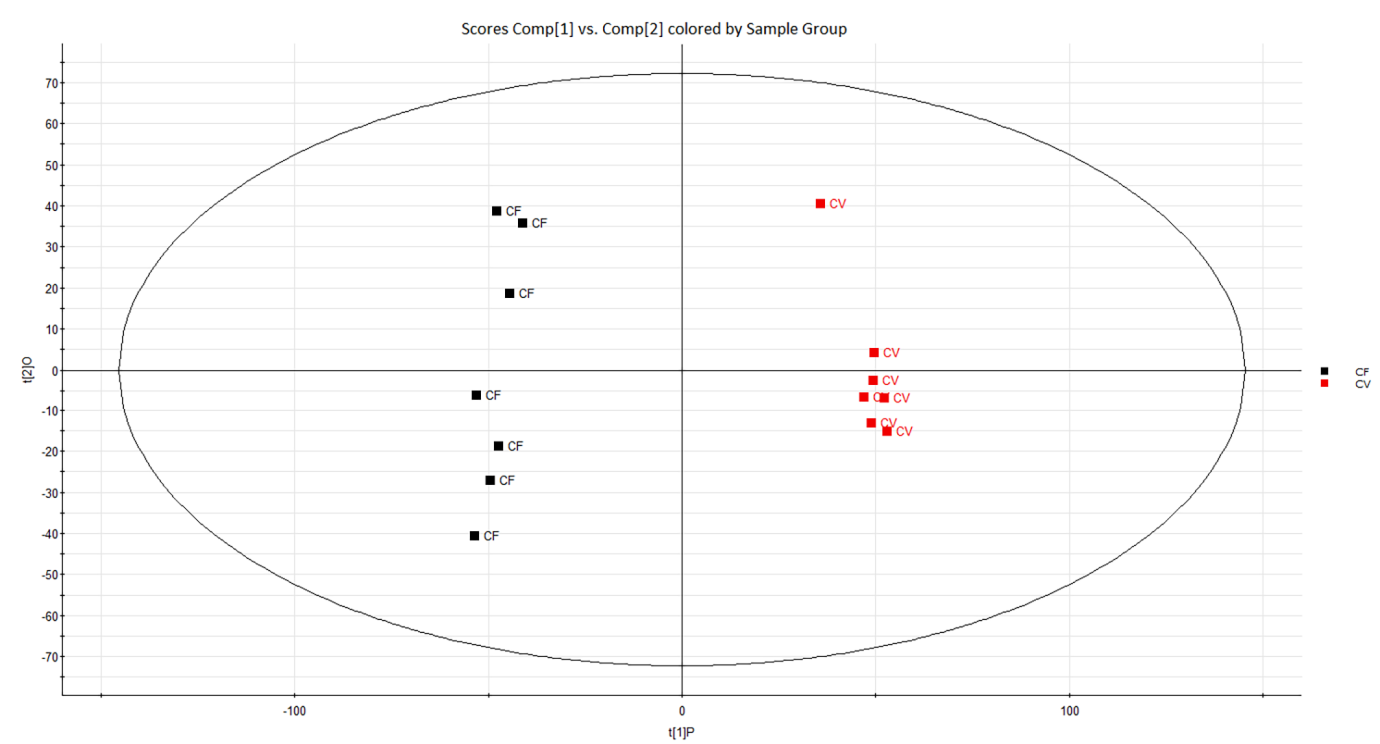


**Fig. S5.** OPLS-DA score plots for pair-wise comparisons between CV and MV (a, b), MV and MF (c, d), and CV and CF groups (e, f) from feces samples. (a, c, e in POS ion mode; b, d, f in NEG ion mode)

(a)
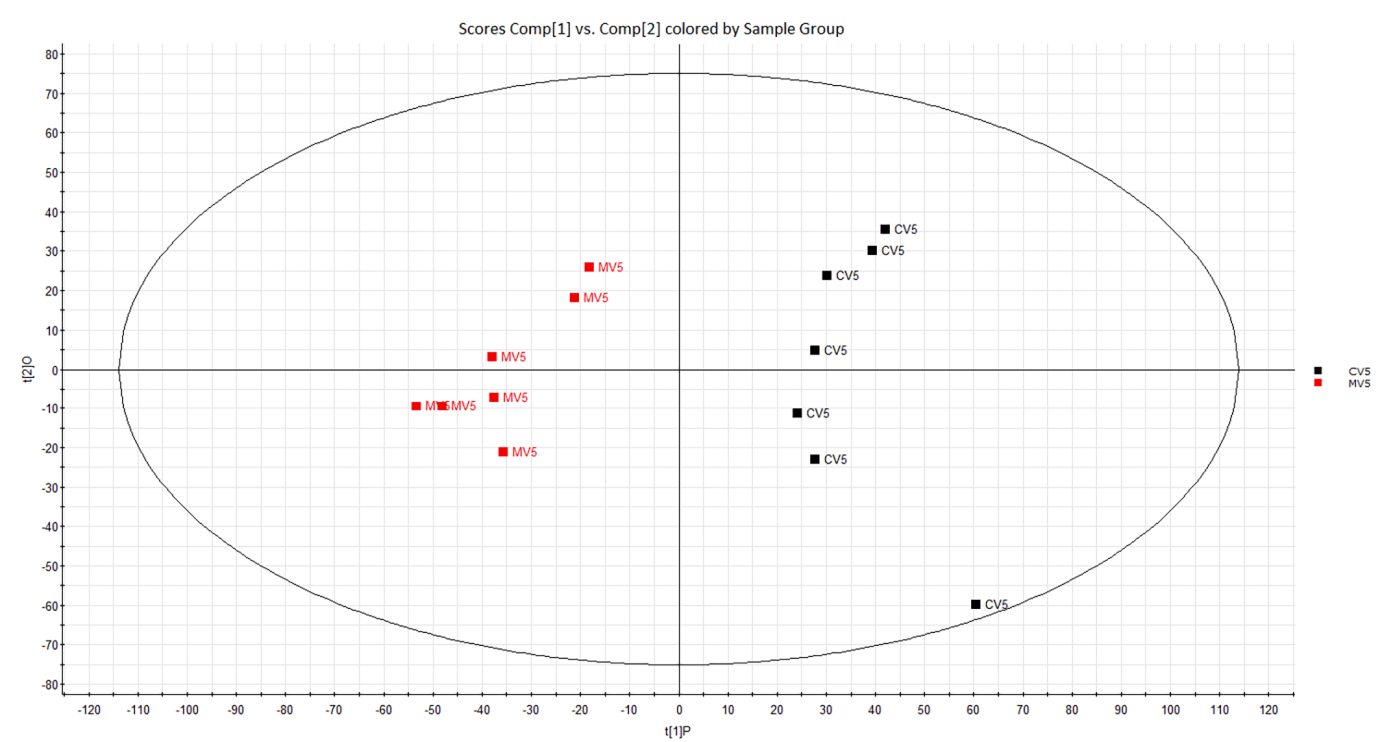


(b)
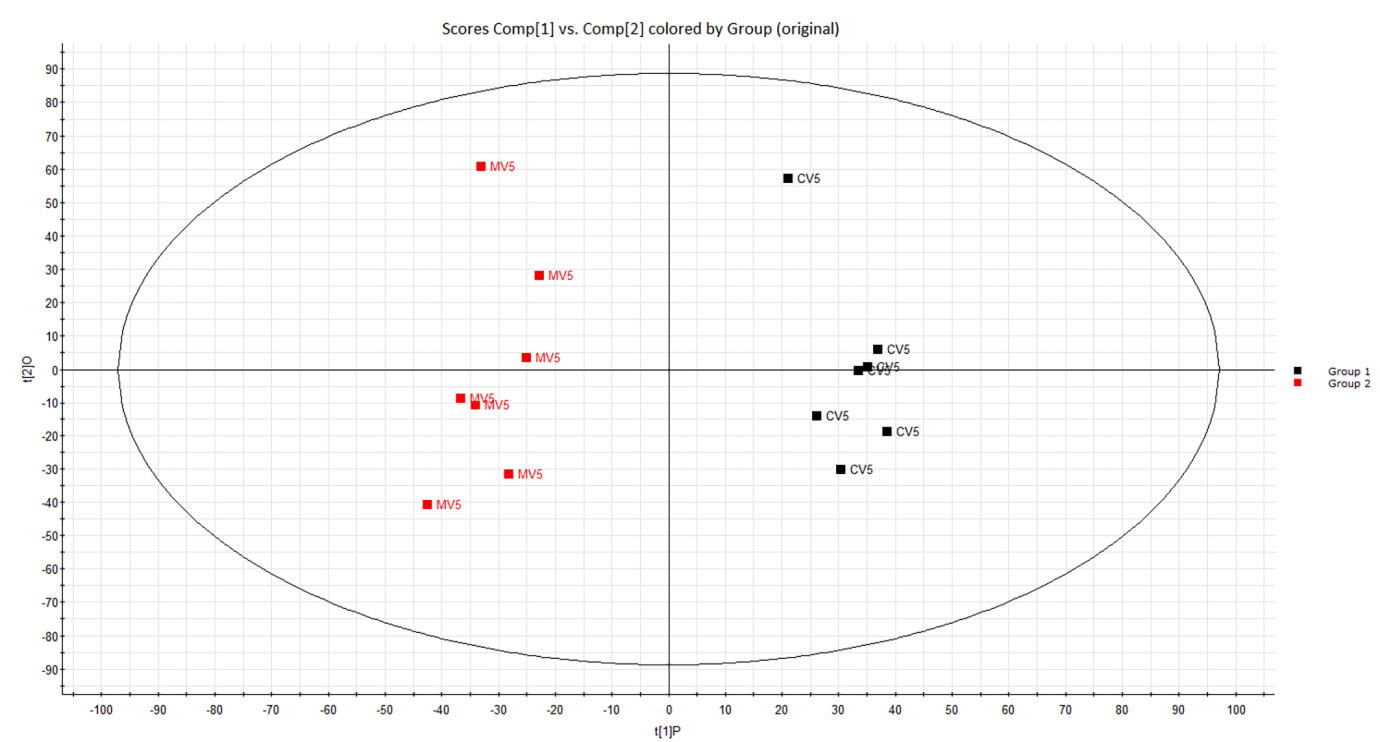


(c)
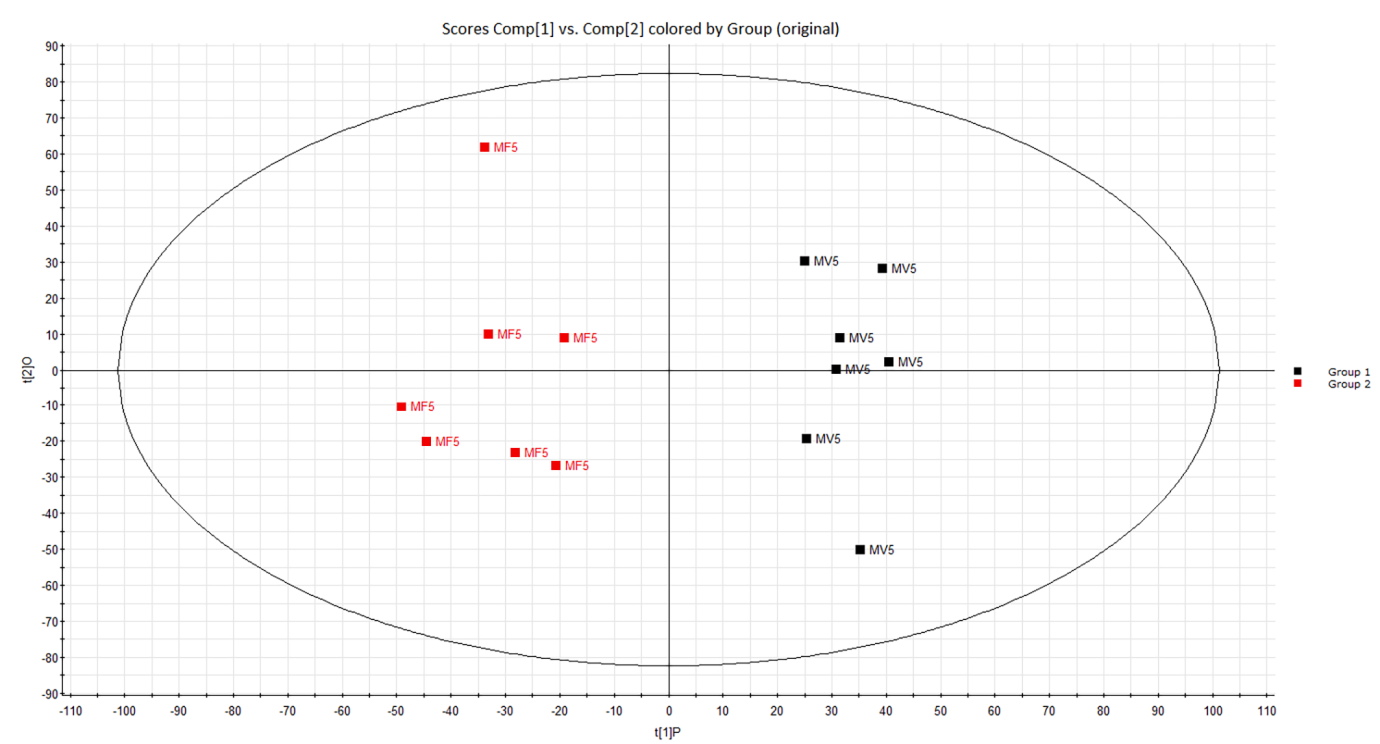


(d)
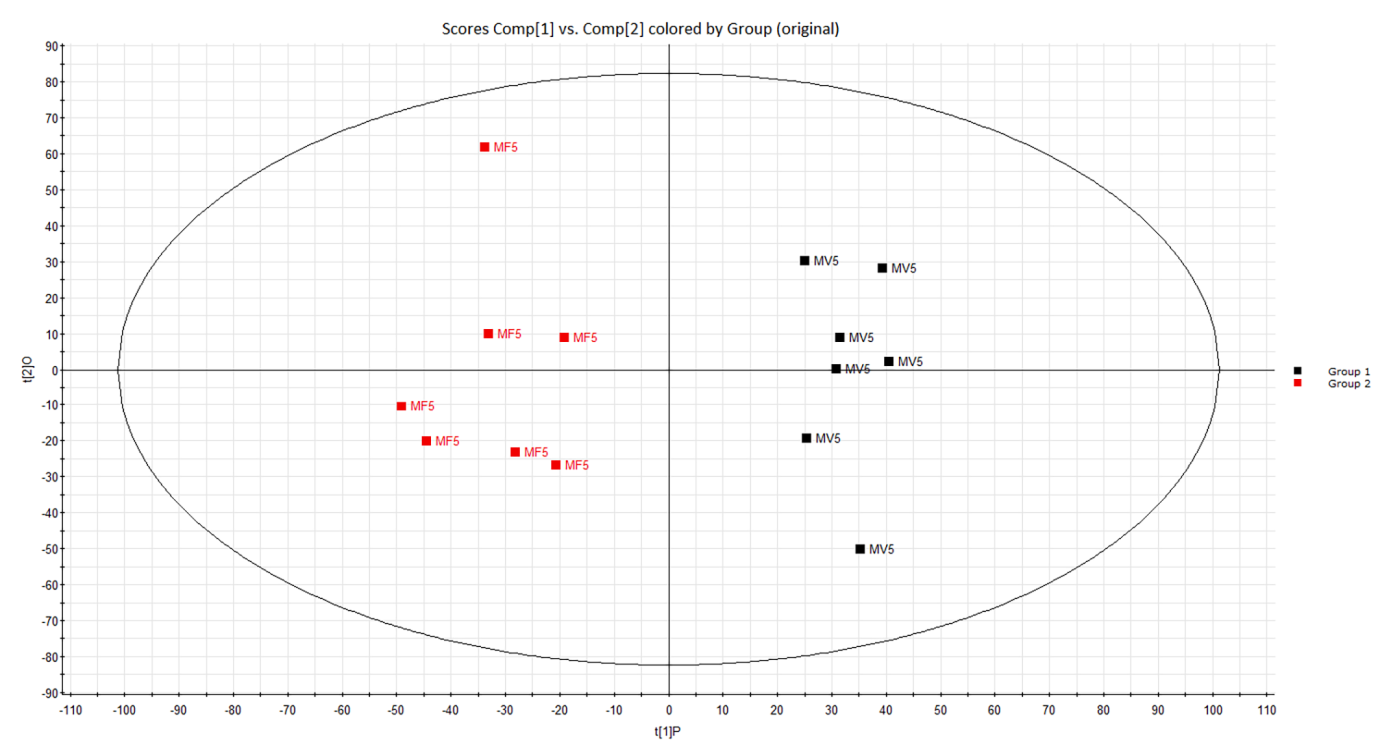


(e)
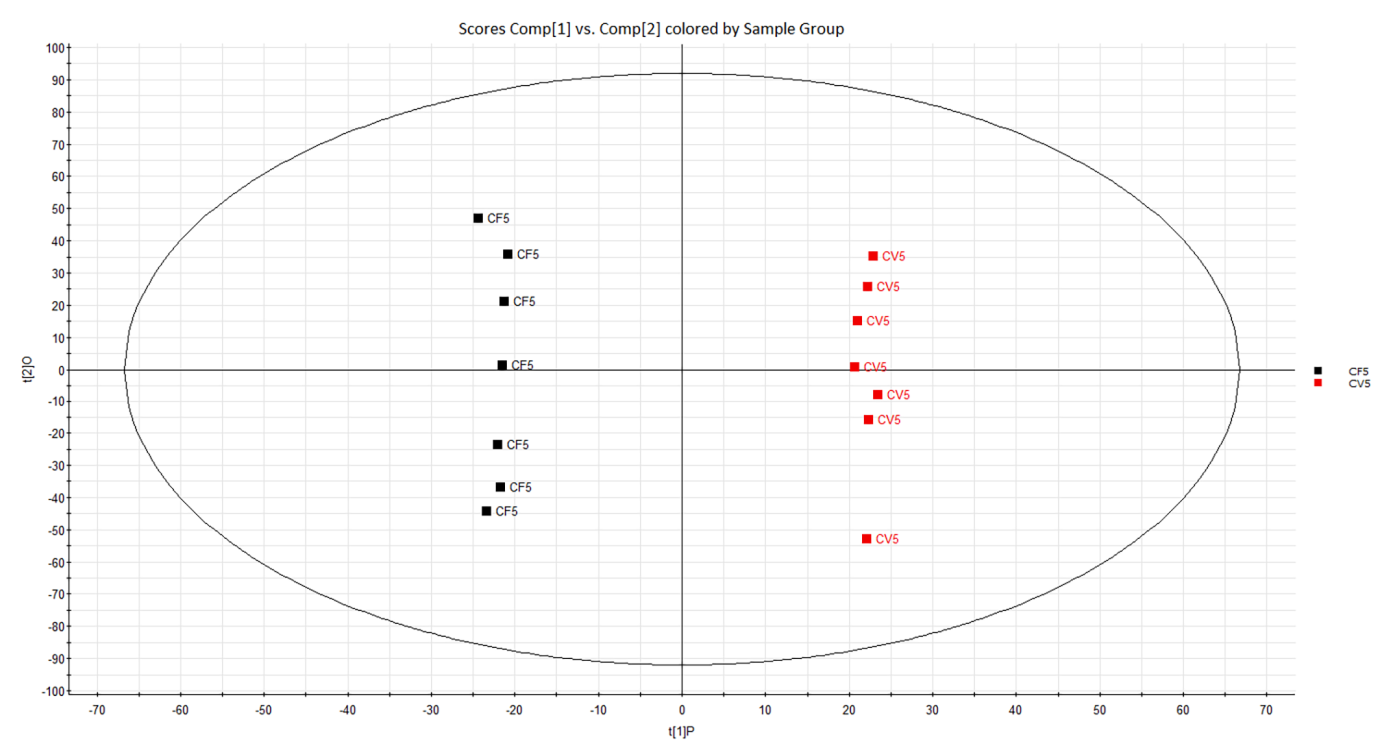


(f)
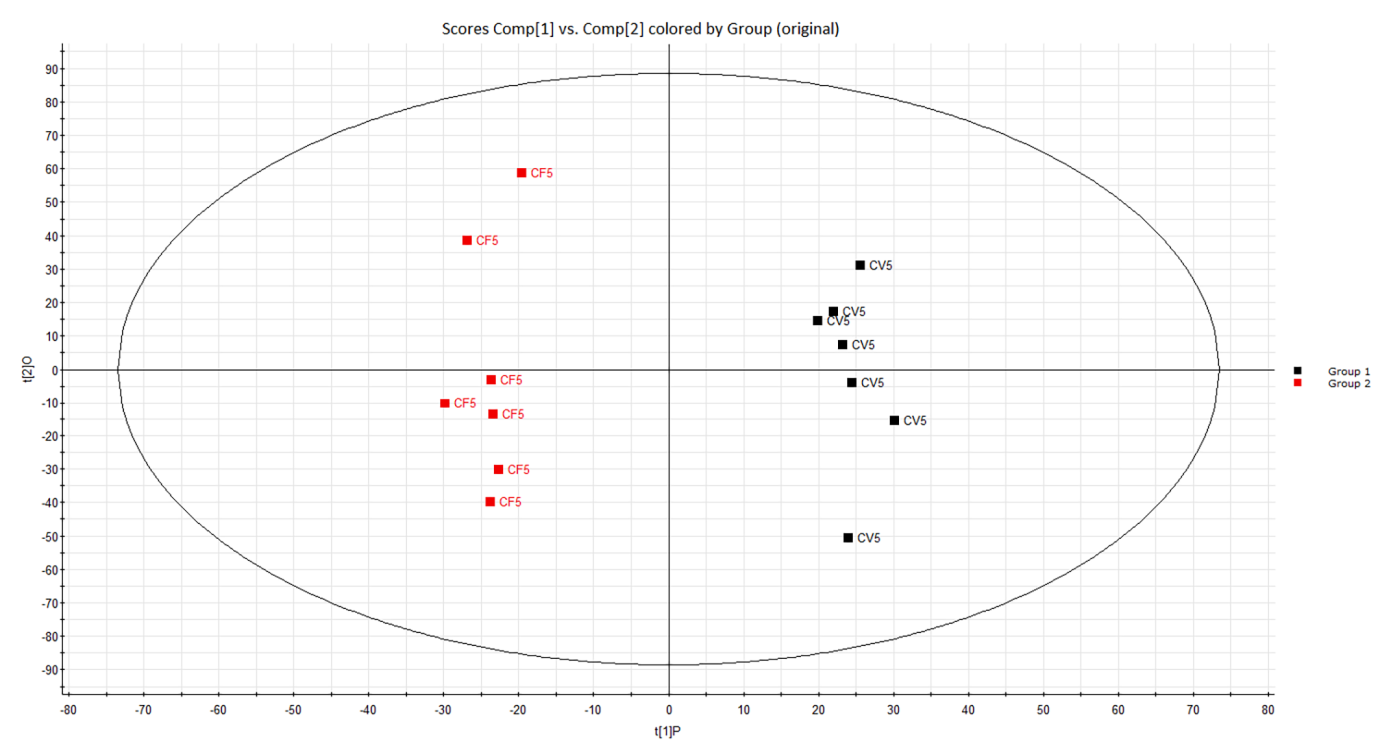


**Fig. S6.** V-plots displaying coefficients *vs.* VIP scores from pair-wise comparisons between CV and MV (a, b), MV and MF (c, d), and CV and CF groups (e, f) from hippocampus samples. (a, c, e in POS ion mode; b, d, f in NEG ion mode)

(a)
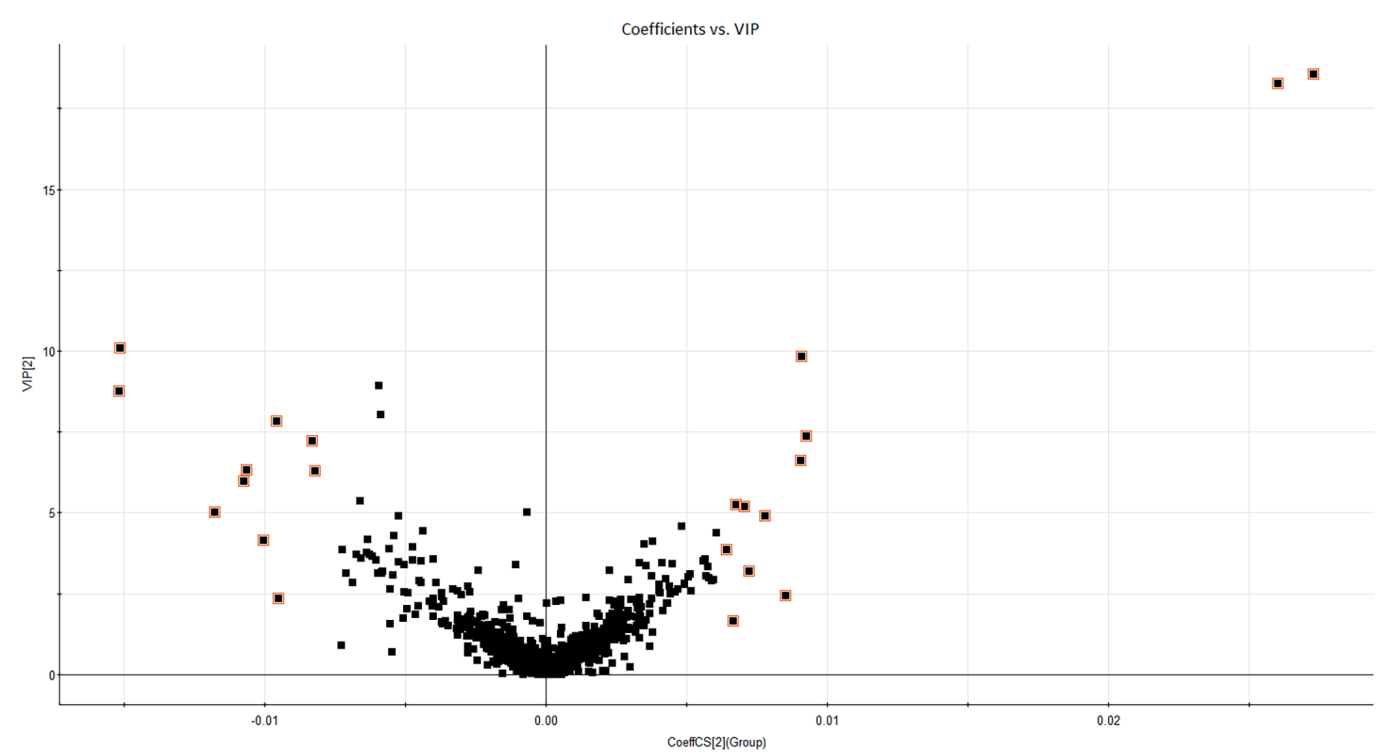


(b)
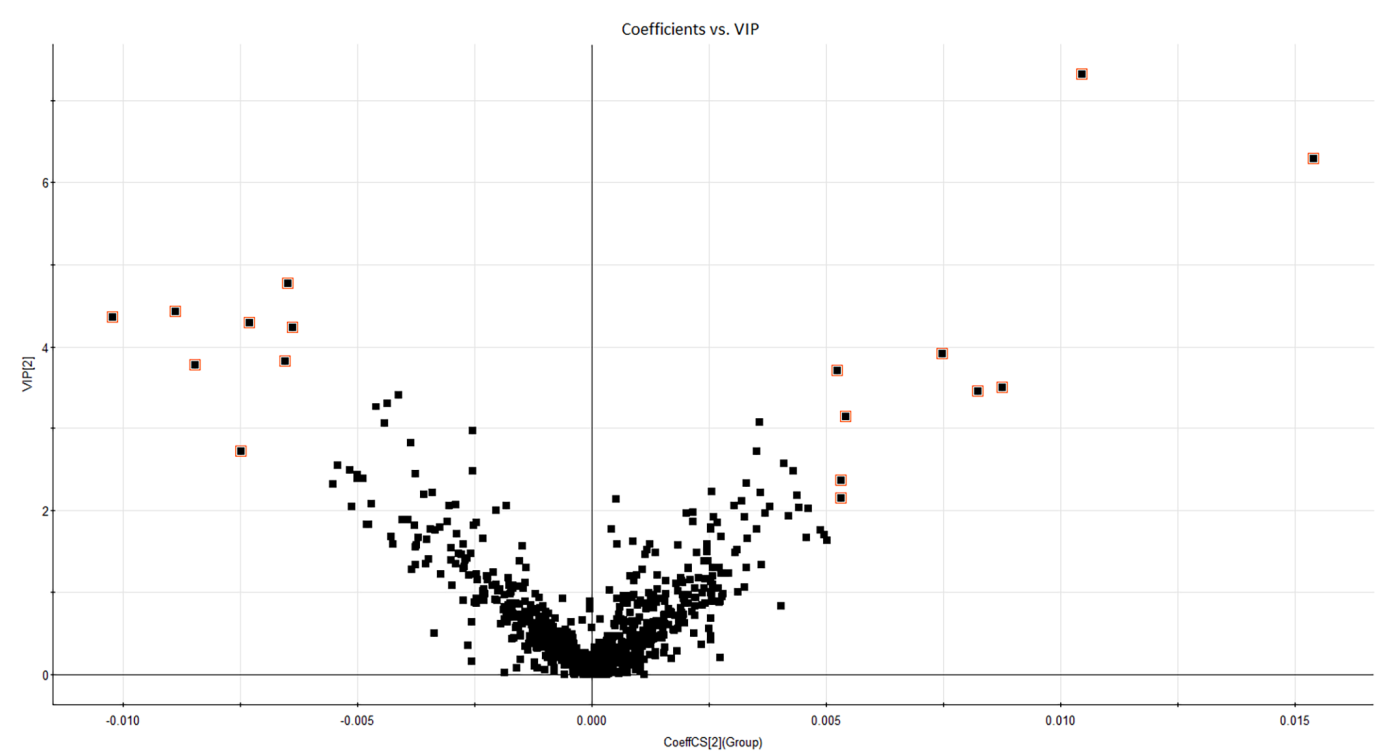


(c)
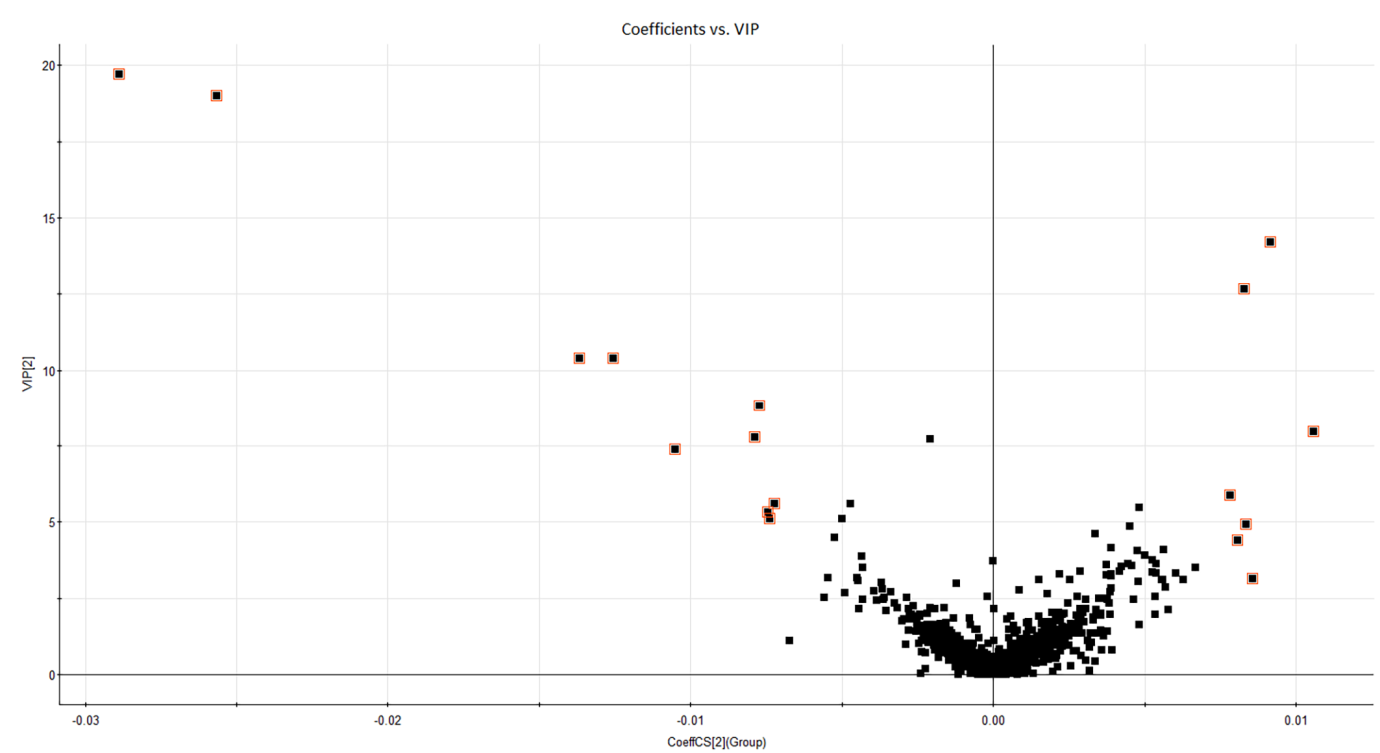


(d)
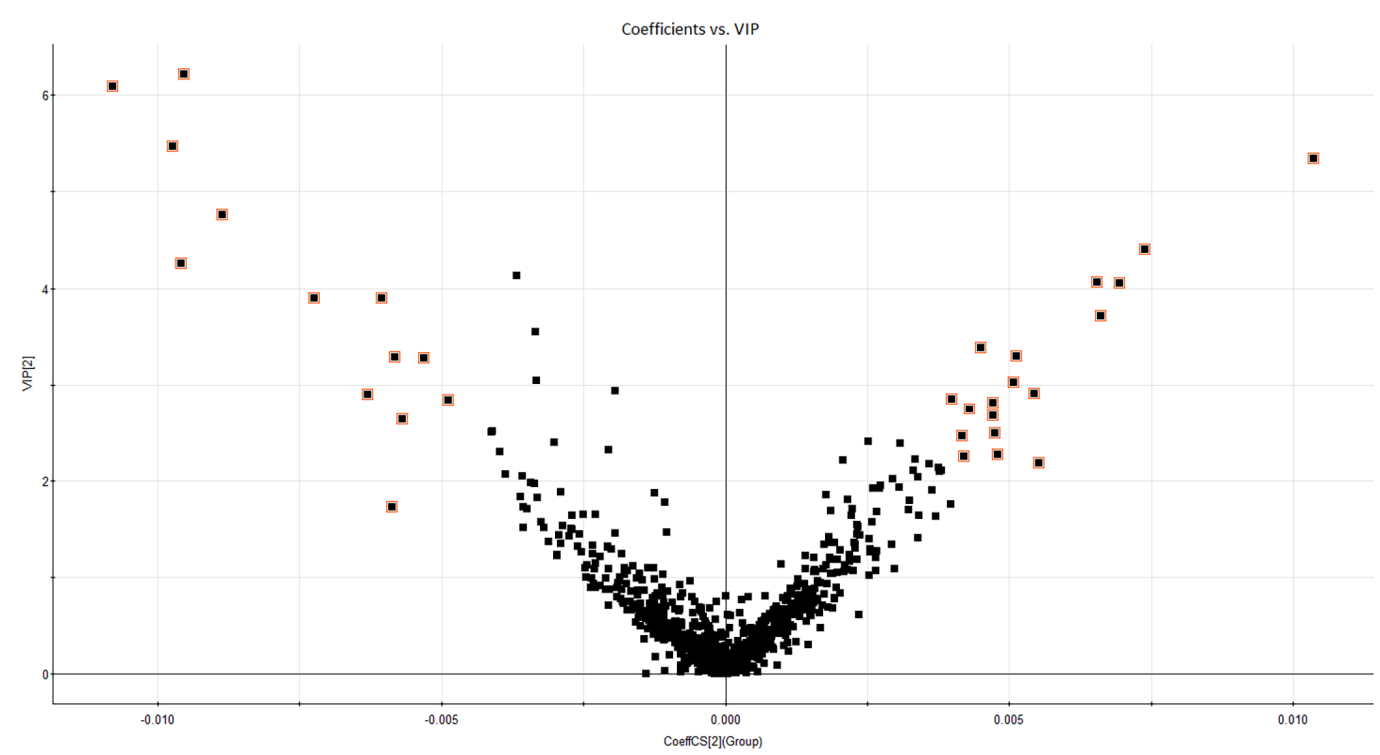


(e)
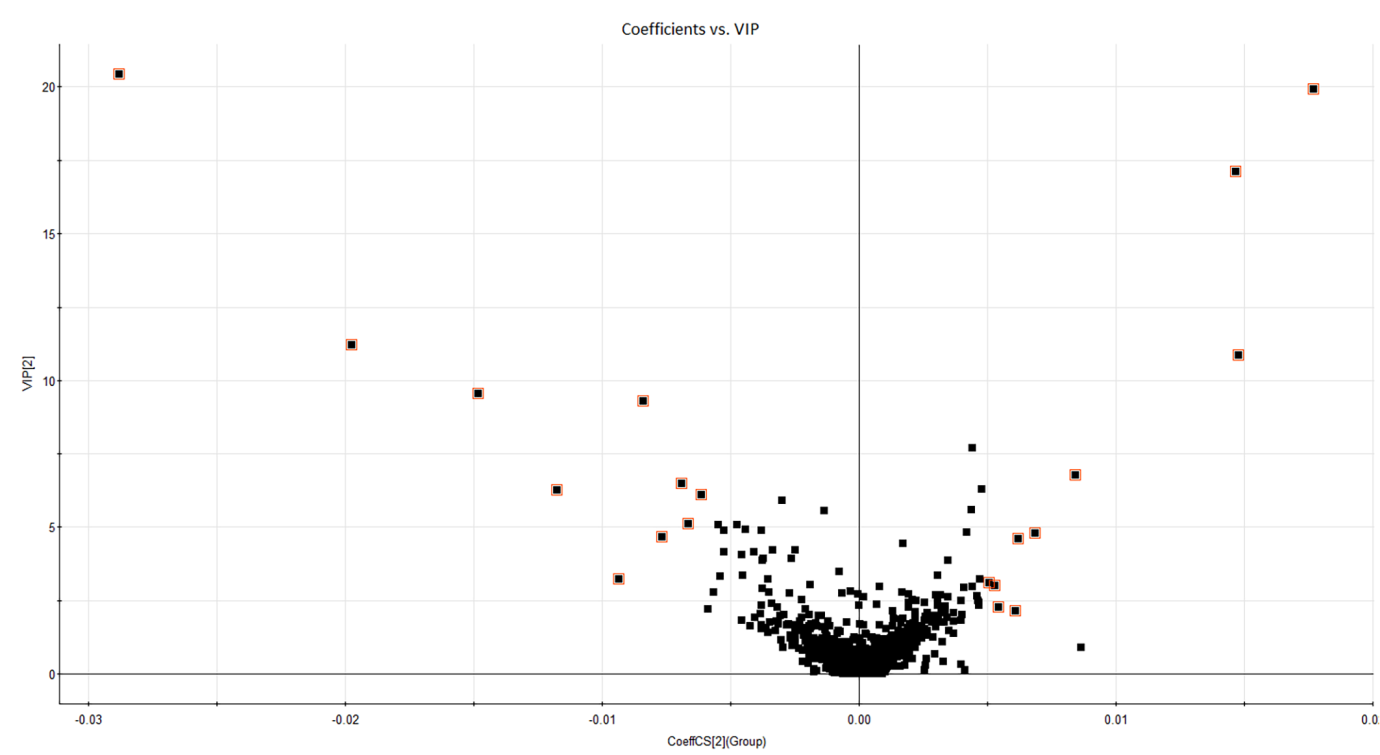


(f)
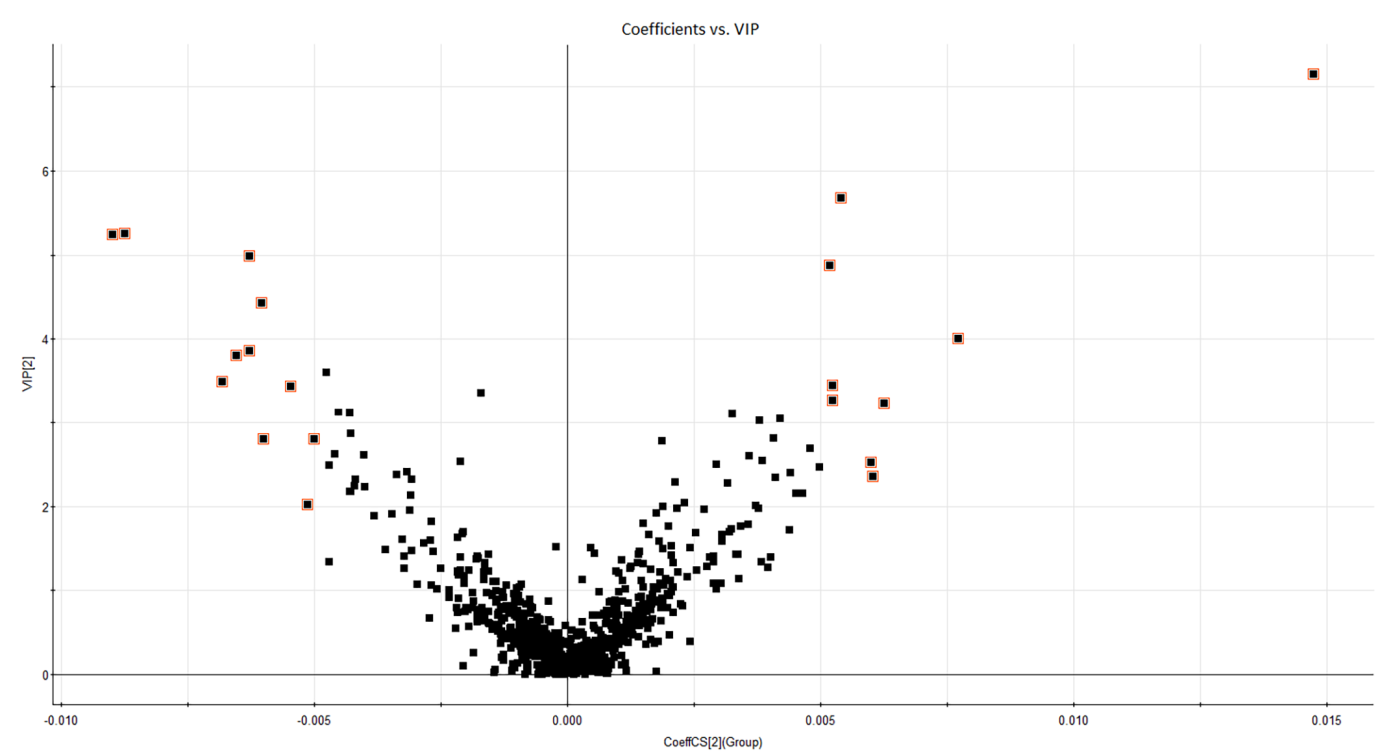


**Fig. S7.** V-plots displaying coefficients *vs.* VIP scores from pair-wise comparisons between CV and MV (a, b), MV and MF (c, d), and CV and CF groups (e, f) from serum samples. (a, c, e in POS ion mode; b, d, f in NEG ion mode)

(a)
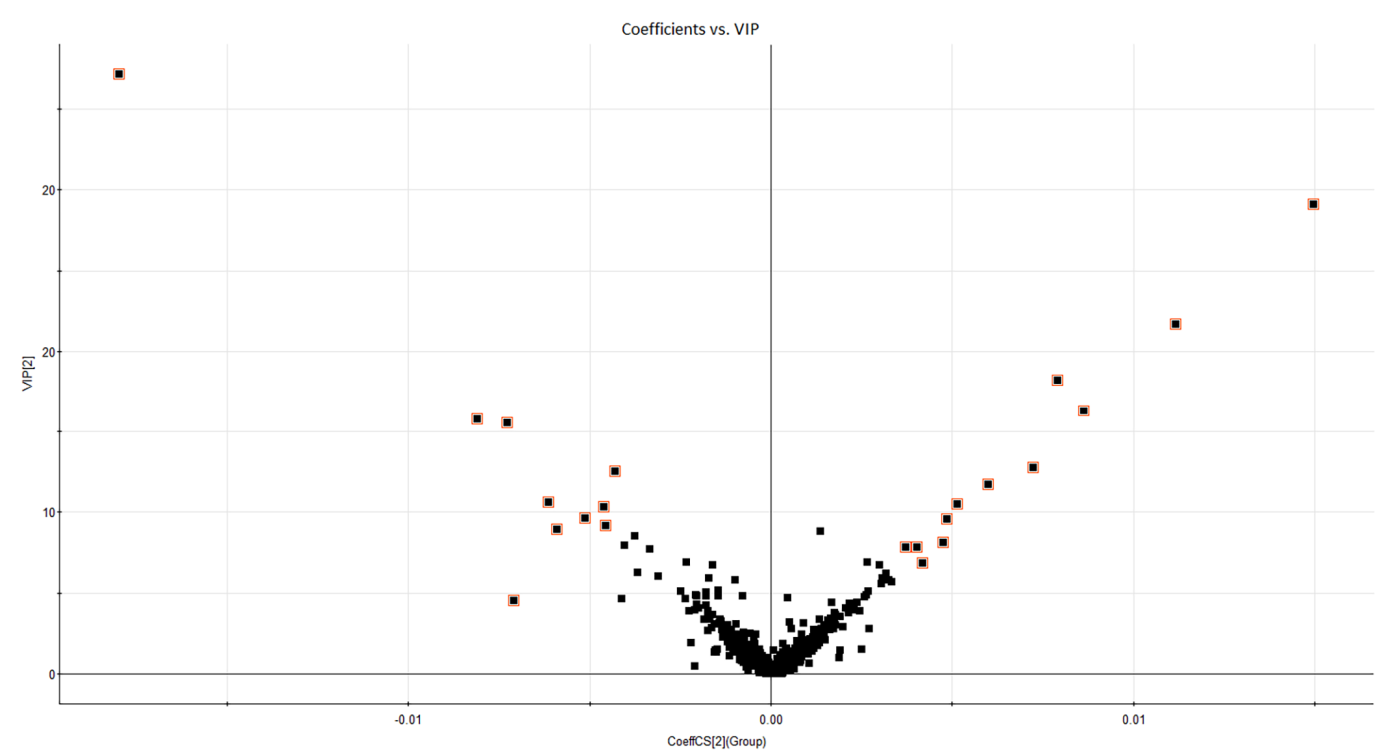


(b)
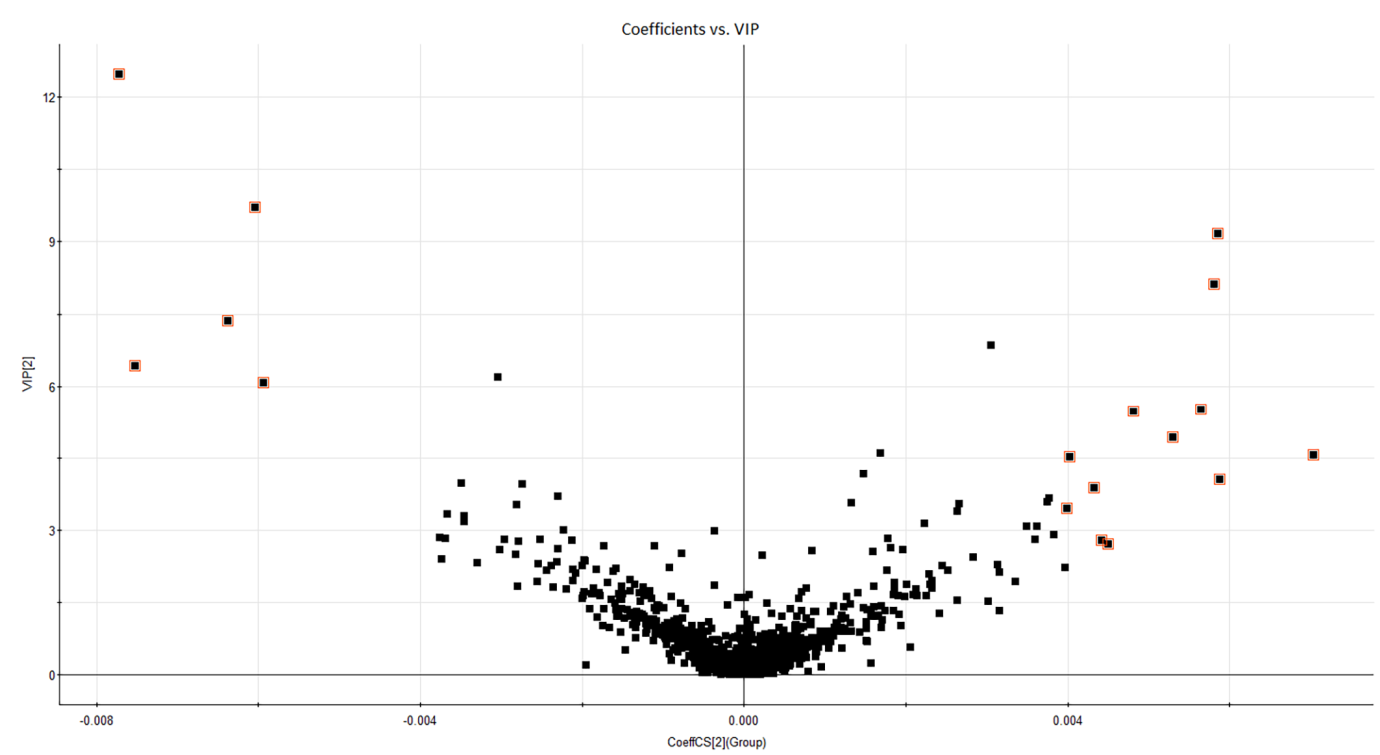


(c)
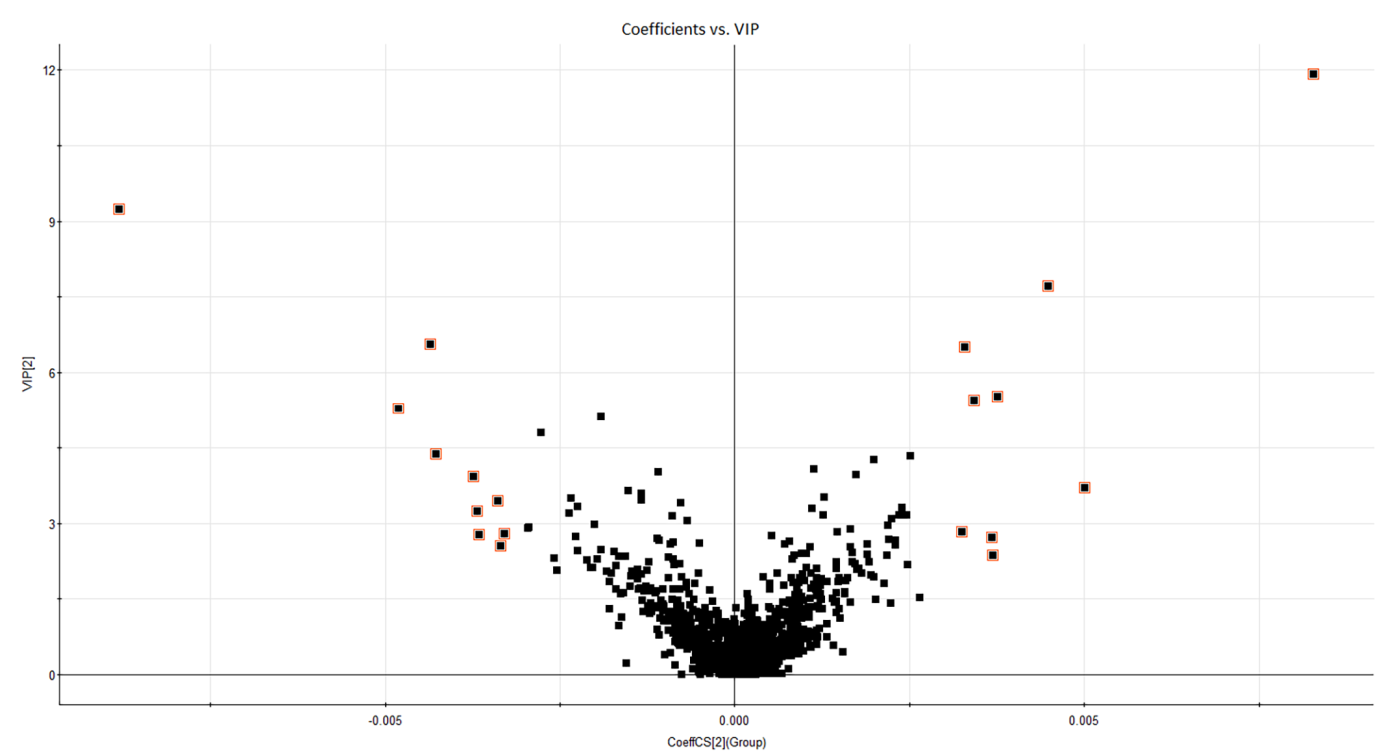


(d)
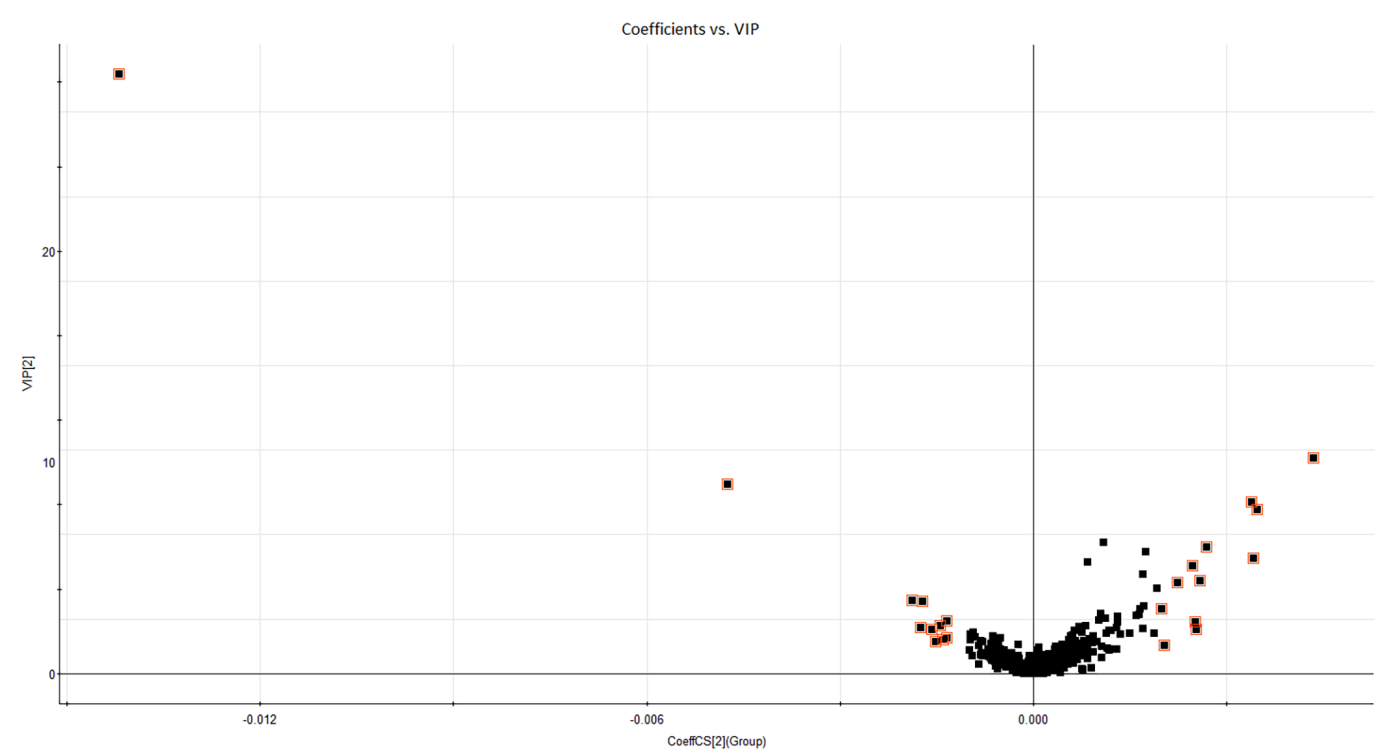


(e)
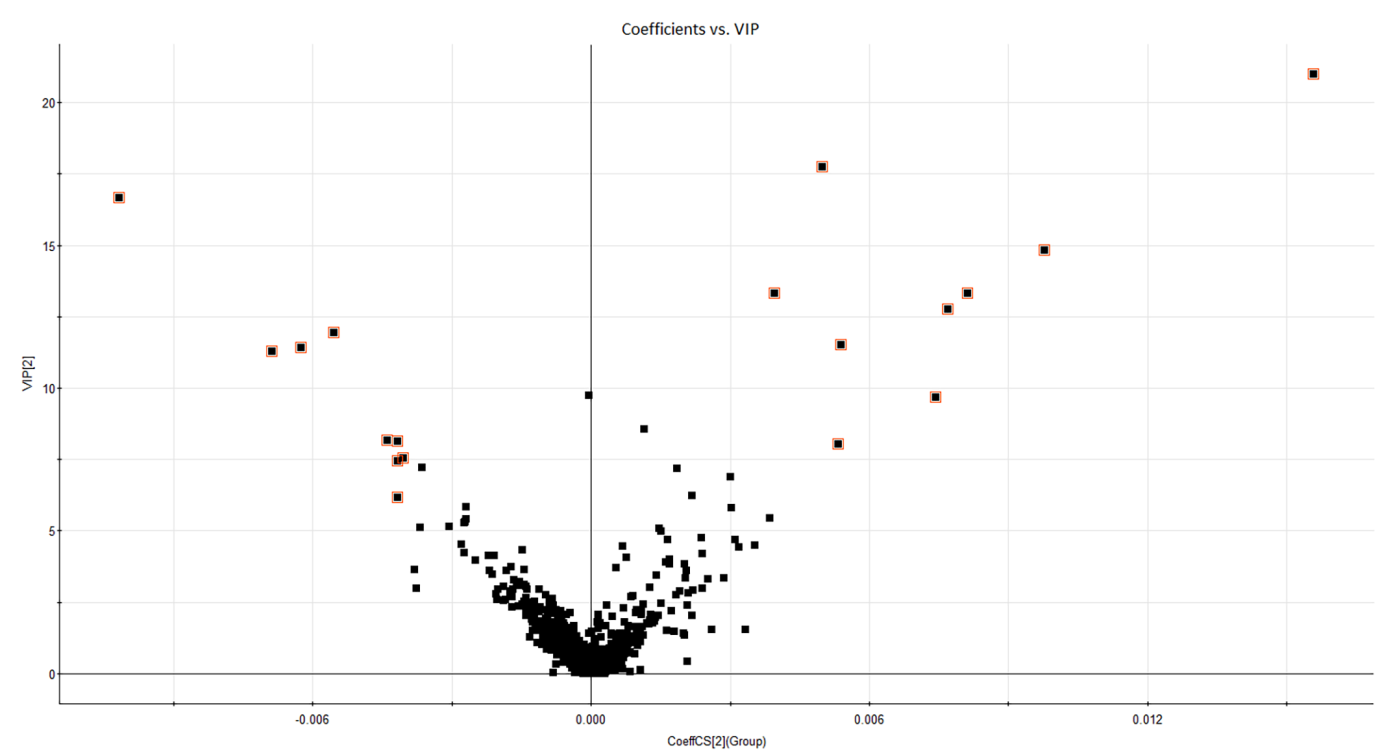


(f)
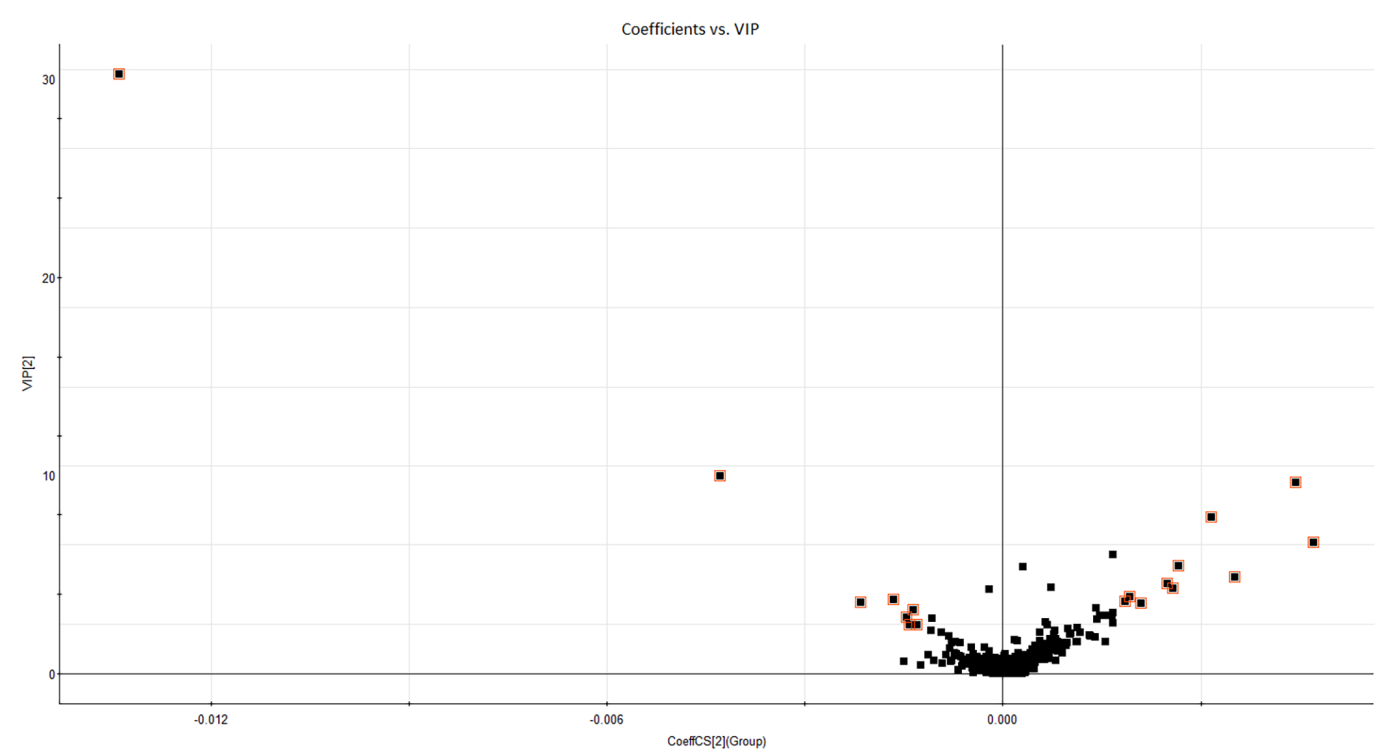


**Fig. S8.** V-plots displaying coefficients *vs.* VIP scores from pair-wise comparisons between CV and MV (a, b), MV and MF (c, d), and CV and CF groups (e, f) from feces samples. (a, c, e in POS ion mode; b, d, f in NEG ion mode)

(a)
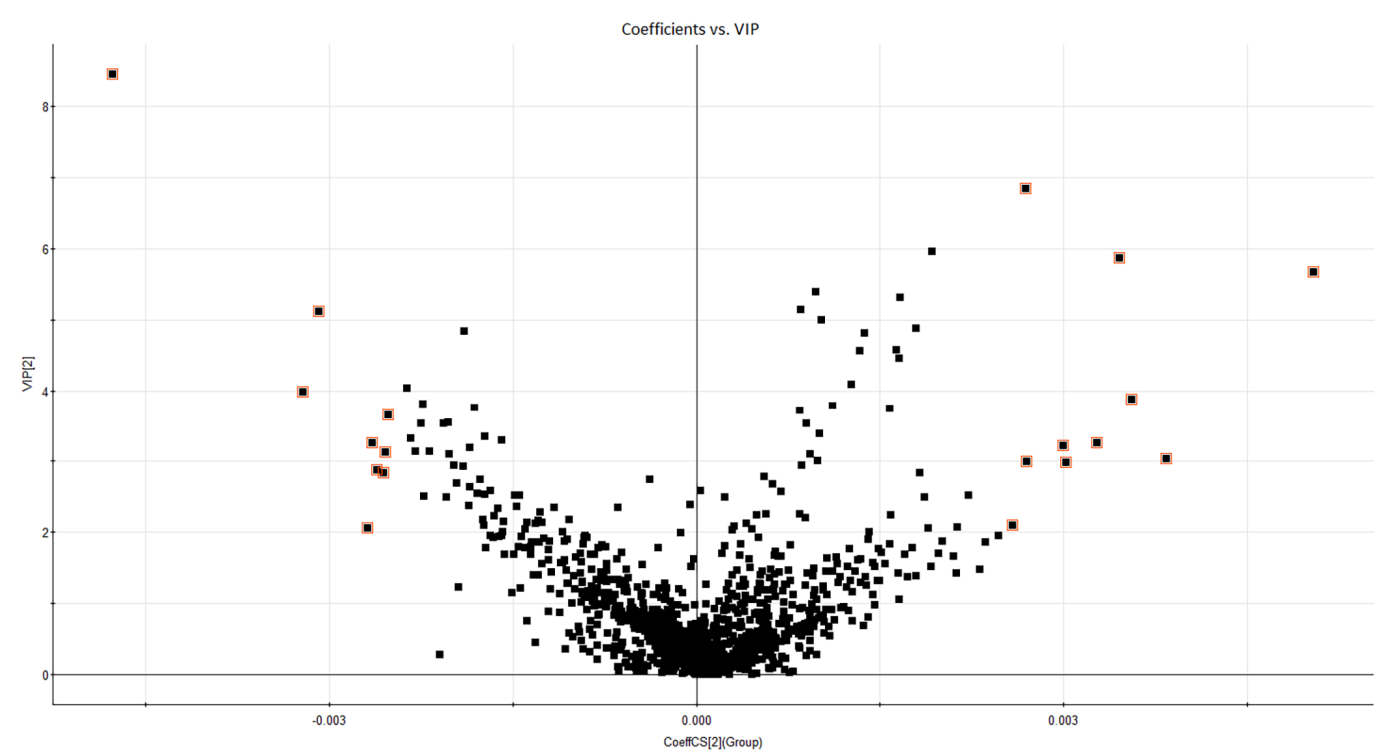


(b)
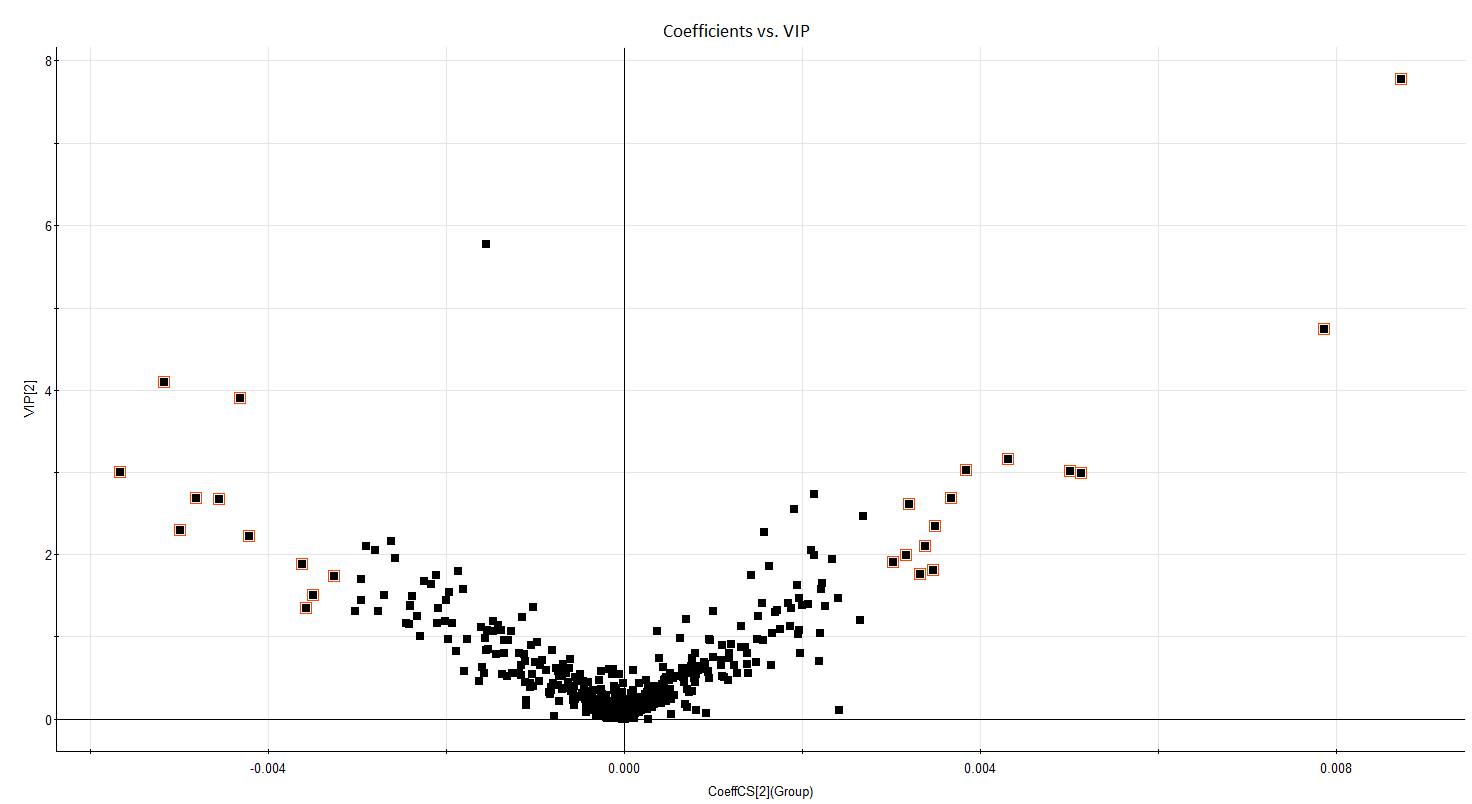


(c)
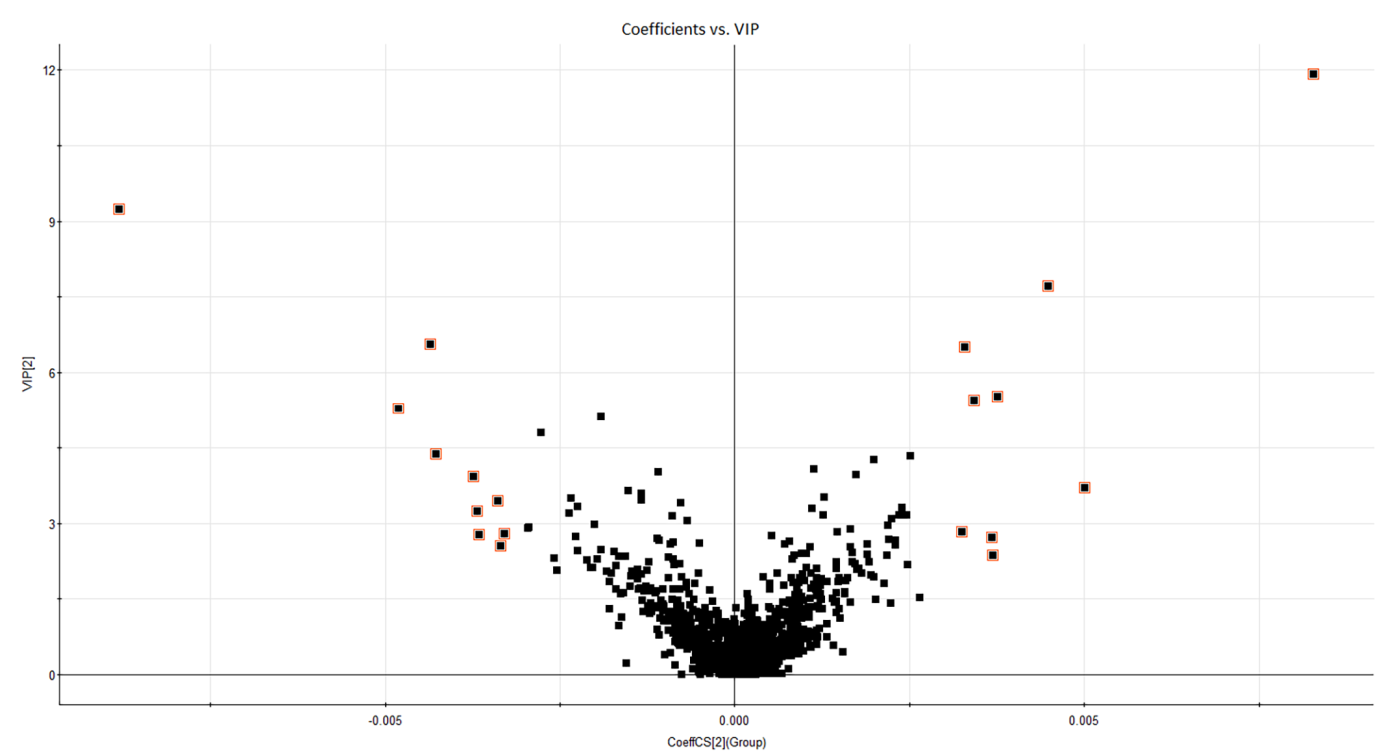


(d)
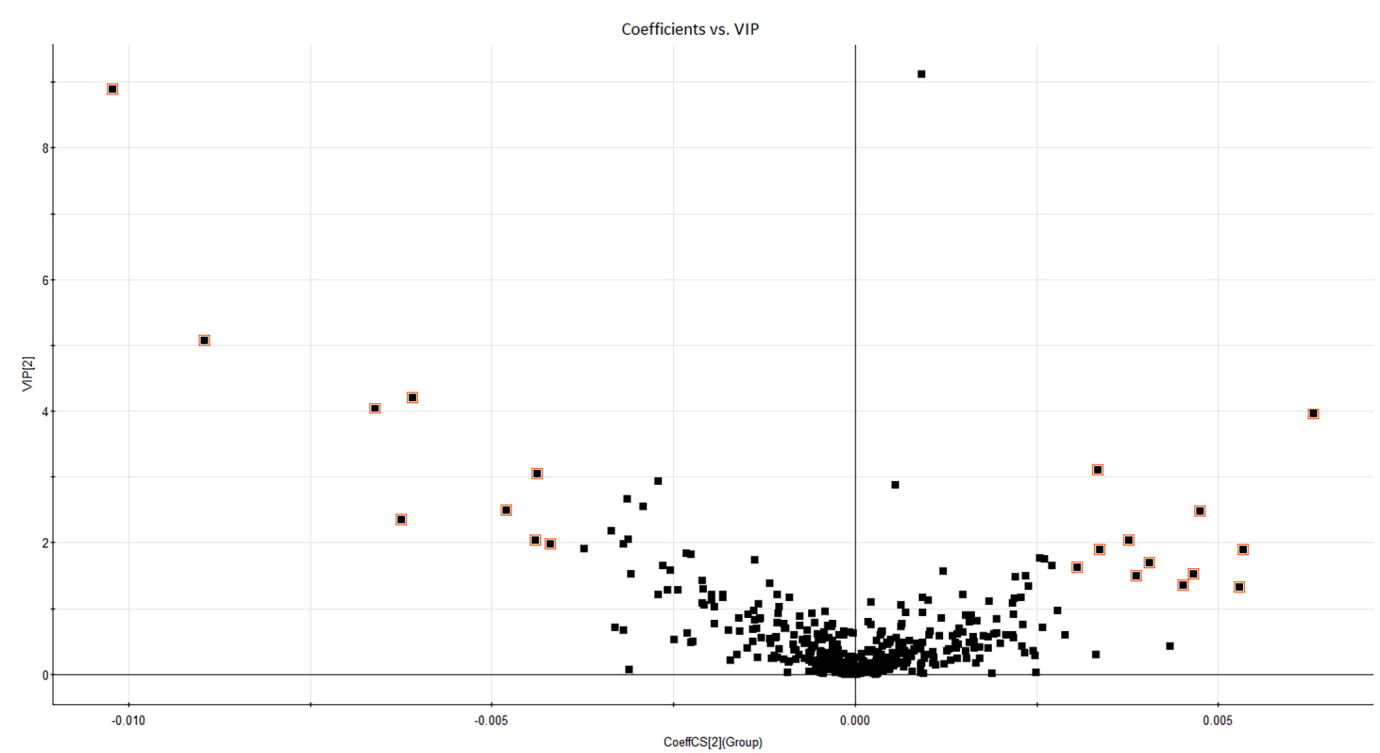


(e)
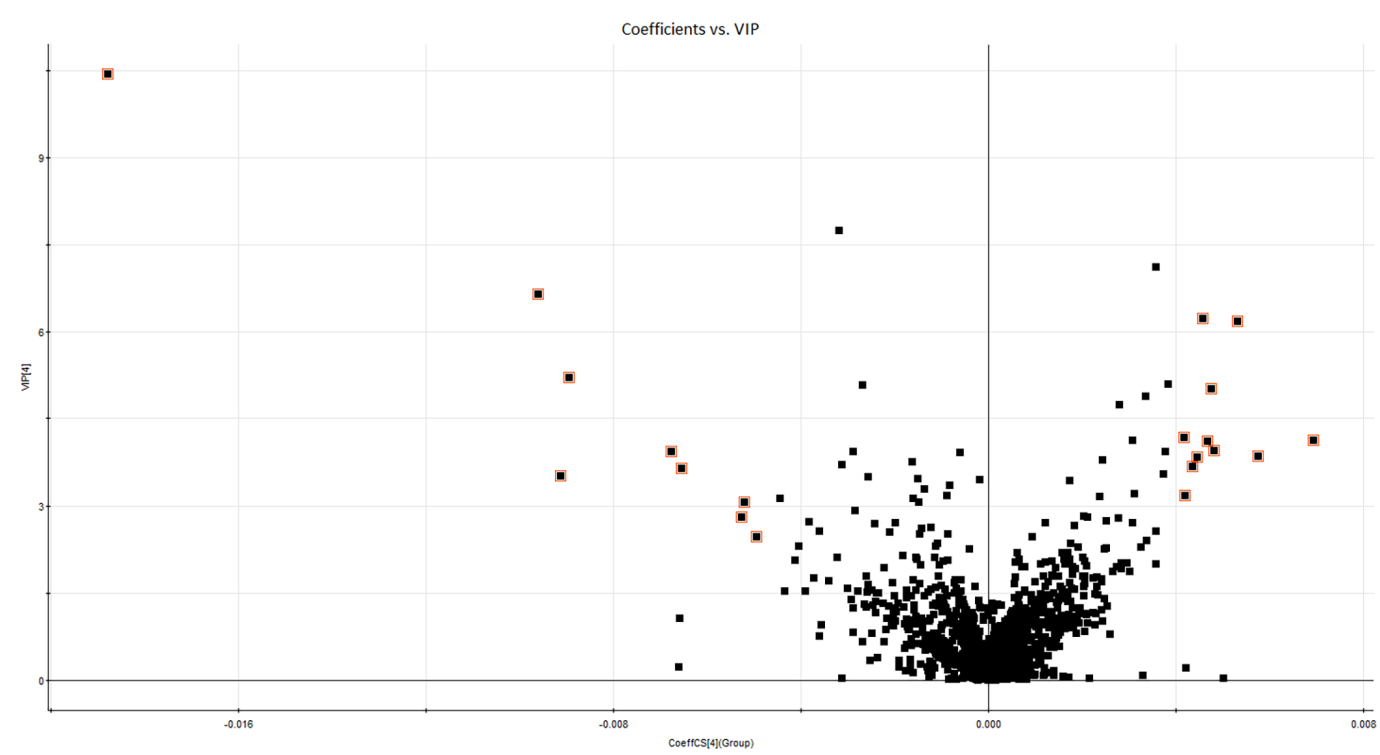


(f)
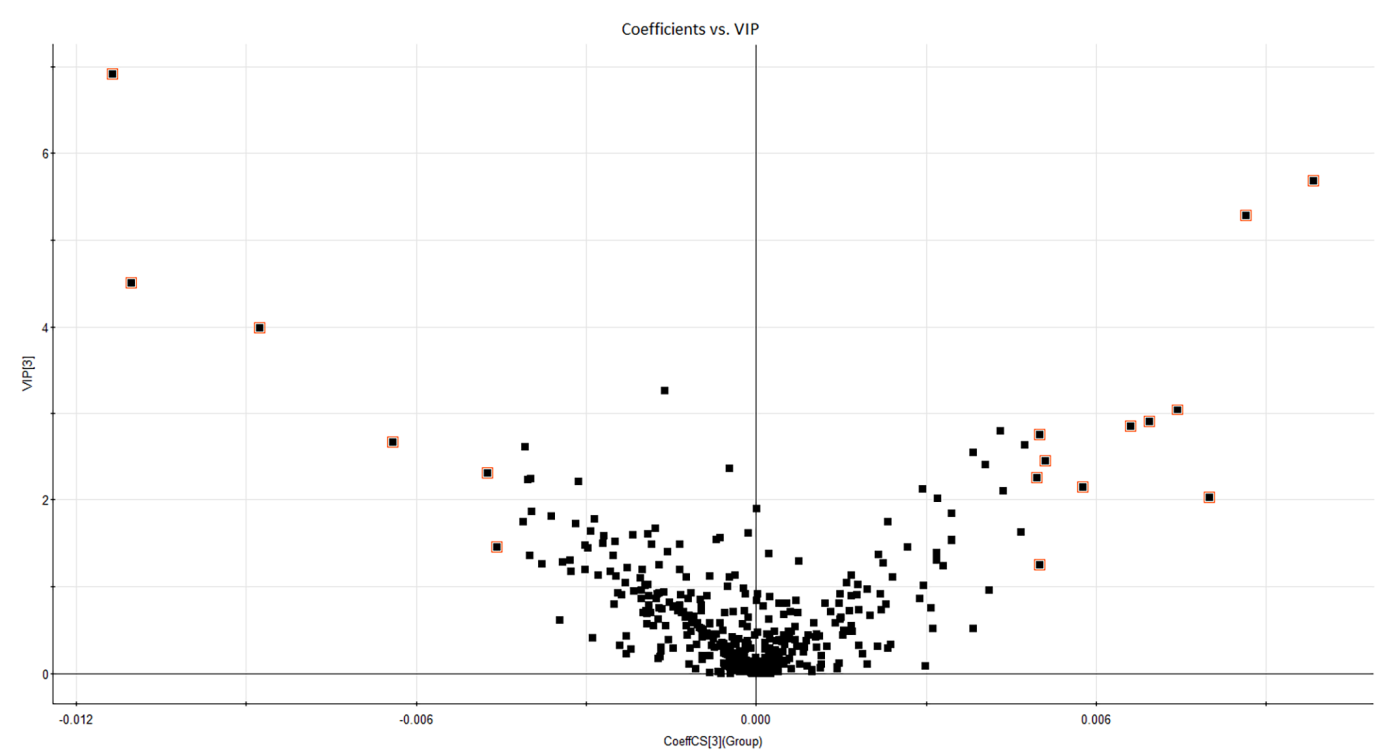

Supplement: Supplementary file 1 — Supplementary information [file 41598_2019_44052_MOESM1_ESM.docx]
